# Supplementary material for: Simplified Intestinal Microbiota to Study Microbe-Diet-Host Interactions in a Mouse Model
Source: Cell Rep. 2019 Mar 26;26(13):3772–3783.e6. doi: 10.1016/j.celrep.2019.02.090 (PMC6444000; doi:10.1016/j.celrep.2019.02.090)
Supplement: Document S1. Tables S1 and S5 [file mmc1.pdf]

Cell Reports, Volume *26*

Supplemental Information

Simplified Intestinal Microbiota to Study

Microbe-Diet-Host Interactions in a Mouse Model

Petia Kovatcheva-Datchary, Saeed Shoaie, Sunjae Lee, Annika Wahlström, Intawat Nookaew, Anna Hallen, Rosie Perkins, Jens Nielsen, and Fredrik Bäckhed

Table S1. Nutritional information of the three mouse diets. Related to STAR methods.

Ingredients (% by weight)	Chow* (Lab Diet 5021)	HF/HS** (Harlan TD.96132)	ZF/HS** (Harlan TD.03314)
**Protein**	**21.0**	**20.9**	**20.0**
**Fat**	**9.4**	**20.2**	**0**
Saturated fatty acids	2.7	8.3	-
Trans fatty acids	-	3.4	-
Monounsaturated fatty acids	2.6	7.1	-
Polyunsaturated fatty acids	4.1	1.4	-
**Carbohydrates*** **	**33.2**	**46.3**	**62.8**
Starch	32.1	16.0	-
Sucrose	0.7	18.3	62.8
Maltodextrin	-	12.0	-
Glucose	0.2	-	-
Fructose	0.2	-	-
**Neutral detergent fiber**	**14.8**	**4.0**	**11.0**
Hemicellulose	10.2	-	-
Cellulose	4.6 ****	4.0	11.0

*Natural ingredient diet; **Purified diet; ***Available carbohydrates; ****Cellulose and lignin.

Table S5. Oligonucleotides, related to KEY RESOURCES TABLE

REAGENT or RESOURCE	SOURCE	IDENTIFIER
Oligonucleotides		
AM1	Sigma-Aldrich	CAGCACGTGAAGGTGGGGAC
AM2	Sigma-Aldrich	CCTTGCGGTTGGCTTCAGAT
Bt1F	Sigma-Aldrich	ATAGCCTTTCGAAAGRAAGAT
Bt1R	Sigma-Aldrich	CCAGTATCAACTGCAATTTTA
Bif164F	Sigma-Aldrich	GGGTGGTAATGCCGGATG
BiADO-2	Sigma-Aldrich	CGAAGGGCTTGCTCCCAGT
AERO-F	Sigma-Aldrich	CTTTCAGCAGGGAAGAGTCAA
AERO-R	Sigma-Aldrich	AGCCATGCACCACCTGTATGG
DSV691-F	Sigma-Aldrich	CCGTAGATATCTGGAGGAACATCAG
DSV826-R	Sigma-Aldrich	ACATCTAGCATCCATCGTTTACAGC
EhalF	Sigma-Aldrich	GCGTAGGTGGCAGTGCAA
EhalR	Sigma-Aldrich	GCACCGRAGCCTATACGG
RrecF	Sigma-Aldrich	GCGGTRCGGCAAGTCTGA
Rrec630mR	Sigma-Aldrich	CCTCCGACACTCTAGTMCGAC
g-Prevo-F	Sigma-Aldrich	CACRGTAAACGATGGATGCC
g-Prevo-R	Sigma-Aldrich	GGTCGGGTTGCAGACC
RrecRi630F	Sigma-Aldrich	GTCATCTAGAGTGTCGGAGG
Erec870R	Sigma-Aldrich	AGTTTYATTCTTGCGAACG
Rflbr730F	Sigma-Aldrich	GGCGGCYTRCTGGGCTTT
Clep866mR	Sigma-Aldrich	CCAGGTGGATWACTTATTGTGTTAA
UniF	Sigma-Aldrich	GTGSTGCAYGGYYGTCGTCA
UniR	Sigma-Aldrich	ACGTCRTCCMCNCCTTCCTC
27F	Sigma-Aldrich	GTTTGATCCTGGCTCAG
1492 R	Sigma-Aldrich	CGGCTACCTTGTTACGAC
