# Supplementary material for: Simplified Intestinal Microbiota to Study Microbe-Diet-Host Interactions in a Mouse Model
Source: Cell Rep. 2019 Mar 26;26(13):3772–3783.e6. doi: 10.1016/j.celrep.2019.02.090 (PMC6444000; doi:10.1016/j.celrep.2019.02.090)
Supplement: Document S2. Article plus Supplemental Information [file mmc5.pdf]

Cell Reports

Resource

Simplified Intestinal Microbiota to Study Microbe-Diet-Host Interactions in a Mouse Model

Graphical Abstract

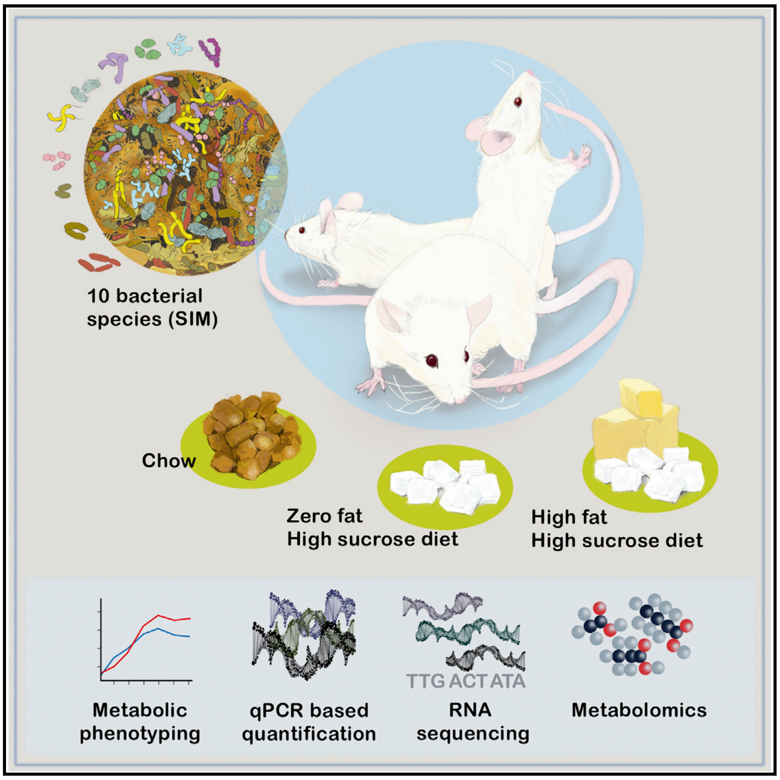

Highlights

- Mice are colonized with ten bacterial strains to create a simple human microbiota model
- Dietary changes alter colonization patterns of the simplified intestinal microbiota (SIM)
- SIM-diet interactions affect some circulating metabolites in the host
- The SIM affects host metabolism in a diet-specific manner

Authors

Petia Kovatcheva-Datchary, Saeed Shoaie, Sunjae Lee, ..., Rosie Perkins, Jens Nielsen, Fredrik Bäckhed

Correspondence

fredrik.backhed@wlab.gu.se

In Brief

Kovatcheva-Datchary et al. develop a mouse model colonized with a simplified intestinal microbiota (SIM) to investigate the microbe-microbe and host-microbe interactions in the mammalian gut, focusing on host metabolism. They combine dietary interventions and different omics approaches to show the potential of the SIM model to study the microbe-diet-host interplay.

Kovatcheva-Datchary et al., 2019, Cell Reports *26*, 3772–3783
March 26, 2019 Crown Copyright © 2019
https://doi.org/10.1016/j.celrep.2019.02.090

CellPress

OPEN
ACCESS
CellPress

Cell Reports
Resource

Simplified Intestinal Microbiota to Study Microbe-Diet-Host Interactions in a Mouse Model

Petia Kovatcheva-Datchary,[1,6,8] Saeed Shoaie,[2,8] Sunjae Lee,[2] Annika Wahlström,[1] Intawat Nookaew,[3,7] Anna Hallen,[1] Rosie Perkins,[1] Jens Nielsen,[3,4] and Fredrik Bäckhed[1,5,9,*]

[1]Wallenberg Laboratory, Department of Molecular and Clinical Medicine, University of Gothenburg, Gothenburg, 41345, Sweden
[2]Centre for Host–Microbiome Interactions, Dental Institute, King's College London, SE1 9RT, UK
[3]Department of Biology and Biological Engineering, Chalmers University of Technology, Gothenburg, 41345, Sweden
[4]Novo Nordisk Foundation Center for Biosustainability, Technical University of Denmark, Kongens Lyngby, 2800, Denmark
[5]Novo Nordisk Foundation Center for Basic Metabolic Research, Section for Metabolic Receptology and Enteroendocrinology, Faculty of Health Sciences, University of Copenhagen, Copenhagen, 2200, Denmark
[6]Present address: CAS Key Laboratory of Separation Science for Analytical Chemistry, Scientific Research Center for Translational Medicine, Dalian Institute of Chemical Physics, Chinese Academy of Sciences, Dalian, 116023 China
[7]Present address: Department of Biomedical Informatics, College of Medicine, University of Arkansas for Medical Sciences, Little Rock, AR 72205, USA
[8]These authors contributed equally
[9]Lead Contact
*Correspondence: fredrik.backhed@wlab.gu.se
https://doi.org/10.1016/j.celrep.2019.02.090

SUMMARY

The gut microbiota can modulate human metabolism through interactions with macronutrients. However, microbiota-diet-host interactions are difficult to study because bacteria interact in complex food webs in concert with the host, and many of the bacteria are not yet characterized. To reduce the complexity, we colonize mice with a simplified intestinal microbiota (SIM) composed of ten sequenced strains isolated from the human gut with complementing pathways to metabolize dietary fibers. We feed the SIM mice one of three diets (chow [fiber rich], high-fat/high-sucrose, or zero-fat/high-sucrose diets [both low in fiber]) and investigate (1) how dietary fiber, saturated fat, and sucrose affect the abundance and transcriptome of the SIM community, (2) the effect of microbe-diet interactions on circulating metabolites, and (3) how microbiota-diet interactions affect host metabolism. Our SIM model can be used in future studies to help clarify how microbiota-diet interactions contribute to metabolic diseases.

INTRODUCTION

The human gut is populated with a dense and complex community of microbes, collectively known as the gut microbiota (Dethlefsen et al., 2007). The dominating phyla in the human gut are Firmicutes, Bacteroidetes, Actinobacteria, Proteobacteria, and Verrucomicrobia, but other phyla, such as Fusobacteria, Cyanobacteria, Lentisphaerae, Spirochaetes, and TM7, are also present (Arumugam et al., 2011; Qin et al., 2010). The gut microbiota is associated with many essential functions for host physiology (Sommer and Bäckhed, 2013). For example, a key function of microbes in the gut is to process complex carbohydrates that cannot be digested by host enzymes (Sonnenburg and Bäckhed, 2016) into short-chain fatty acids (SCFAs), primarily acetate, propionate, and butyrate, and organic acids such as succinate and lactate (Cummings and Macfarlane, 1991; Koh et al., 2016; Macfarlane and Macfarlane, 2012). These metabolites are not only essential for the growth and cellular function of certain microbes in the gut (Cummings and Macfarlane, 1991; Fischbach and Sonnenburg, 2011; Koh et al., 2016) but also affect host physiology (Koh et al., 2016; Zhao et al., 2018).

Although the production of SCFAs resulting from the bacterial fermentation of fiber-rich diets is generally associated with beneficial metabolic effects, the increased energy harvest has also been proposed to contribute to diet-induced obesity in mice (Koh et al., 2016). Furthermore, the gut microbiota produces other metabolites that are influenced by the diet, many of which are likely to play a role in host physiology (Koh et al., 2016; Makki et al., 2018; Zmora et al., 2019). Studies to delineate the interactions between gut bacteria, diet, and host metabolism are challenging because (1) of the complexity and high inter-individual variability of human gut microbiota, (2) bacteria interact in complex food webs in concert with the host, and (3) the microbiota consists mainly of non-sequenced members, thus limiting interpretation from metagenomic and metatranscriptomic analyses. One approach to overcome these issues is to use gnotobiotic animals colonized with a simplified intestinal microbial community consisting of well-characterized bacteria from humans. A recent example of such a model used rats colonized with eight bacterial species from the human gut to investigate how the microbiota composition changes in response to dietary challenges (Becker et al., 2011). Others have used mice colonized with a defined community of human bacteria to investigate microbe-microbe interactions (McNulty et al., 2013; Rey et al., 2013) or the interactions between microbiota, dietary fiber, and the

 Crown Copyright © 2019
This is an open access article under the CC BY license (http://creativecommons.org/licenses/by/4.0/).

Table 1. Phylogenetic and Metabolic Features of the Members of the SIM

SIM Bacterium	Phylum	Metabolic Function	Produced Metabolites	References
Akkermansia muciniphila: DSM 22959	Verrucomicrobia	mucin degradation	acetate, propionate	Derrien et al. (2004)
Bacteroides thetaiotaomicron: ATCC 29148	Bacteroidetes	polysaccharide breakdown; mucin	acetate, propionate, succinate	Martens et al. (2008); Xu et al. (2003)
Bifidobacterium adolescentis: L2-32	Actinobacteria	di- and oligosaccharide breakdown	acetate, lactate	Duranti et al. (2016); Marquet et al. (2009); Pokusaeva et al. (2011)
Collinsella aerofaciens: DSM 3979	Actinobacteria	di- and oligosaccharide breakdown	acetate, lactate, formate, H_2	Flint et al. (2012); Kageyama and Benno (2000)
Desulfovibrio piger: DSM 749	Proteobacteria	sulfate reducer, lactate user	acetate, H_2S	Marquet et al. (2009)
Eubacterium hallii: L2-7	Firmicutes	di- and monosaccharide breakdown; lactate user	butyrate	Duncan et al. (2004b); Scott et al. (2014)
Eubacterium rectale: A1-86	Firmicutes	oligosaccharide breakdown; acetate user	butyrate, lactate, formate, H_2	Duncan and Flint (2008); Duncan et al. (2008)
Prevotella copri: DSM 18205	Bacteroidetes	polysaccharide breakdown	succinate, H_2	Flint et al. (2012); Hayashi et al. (2007); Kovatcheva-Datchary et al. (2015)
Roseburia inulinivorans: A2-194	Firmicutes	oligosaccharide breakdown	butyrate, propionate	Duncan et al. (2006); Scott et al. (2006)
Ruminococcus bromii: L2-63	Firmicutes	polysaccharide breakdown	acetate, formate, H_2	Crost et al. (2018); Ze et al. (2012)

colonic mucus barrier (Desai et al., 2016). However, none of these studies investigated how diet-induced changes in the gut microbiota affect host metabolism in parallel with altered microbial gene expression.

To investigate the effect of microbiota-diet interactions on host metabolism, we developed a gnotobiotic mouse model colonized with ten representatives of the human intestinal microbiota (simplified intestinal microbiota [SIM]). Our selection was based on the following criteria: (1) All members of the dominant phyla in the human gut should be represented. (2) The chosen strains must have been sequenced, thus allowing us to characterize how specific nutrients (fiber, saturated fat, and sucrose) affect the microbial gene expression. (3) The chosen strains must have well-characterized metabolic functions (to digest and/or ferment carbohydrates) and trophic interactions with a range of cross-feeding interactions for dietary conversions in the gut. The main metabolic function and metabolites produced by each SIM bacterial strain in *in vitro* studies are presented in Table 1. Because H_2 is produced during fermentation and accumulation of H_2 decreases the metabolic activity of microbes (Rey et al., 2013), we also included the H_2-consuming sulfate-reducing bacterium *Desulfovibrio piger*.

RESULTS AND DISCUSSION

Colonization Pattern of the SIM Community in Mice on a Chow Diet

We introduced the ten selected bacteria (Table 1) into germ-free (GF) Swiss Webster mice and maintained them for four generations to generate stably colonized SIM mice. We showed that all ten bacteria of the SIM community colonized each region of the gut of SIM mice on a chow diet, although *Eubacterium hallii* was present only at a low density throughout (Figure 1A). Levels of SIM bacteria were higher in the distal part of the gut compared with the small intestine, with the highest levels in the feces of SIM mice (Figure 1A), consistent with the fact that the SIM bacteria were chosen because of their importance for carbohydrate fermentation.

A previous metagenome analysis has shown that all of the SIM bacteria, with the exception of *Roseburia inulinivorans*, are frequent colonizers of the human gut (Qin et al., 2010). We confirmed that the ten taxa of the SIM community were present in feces from two healthy human donors (Figures 1B and 1C). For comparison with SIM mice, we then colonized GF mice with unfractionated microbiota from each of the two human donors for 2 weeks. We showed that all ten SIM bacteria were present in the cecum of chow-fed mice colonized with feces from one of these donors (Figure 1B) and all except *Collinsella aerofaciens* were present in the cecum of chow-fed mice colonized with microbiota from the second donor (Figure 1C). This unsuccessful colonization of *C. aerofaciens* may be explained by the fact that the second donor had low fecal levels of *C. aerofaciens* and that this species must compete with other members of the human microbiota beyond the ten present in the SIM community. However, the overall profiles of the ten SIM bacteria were similar in the SIM mice and the humanized mice on a chow diet, indicating that the SIM community may be a suitable simplified model of the human gut microbiota.

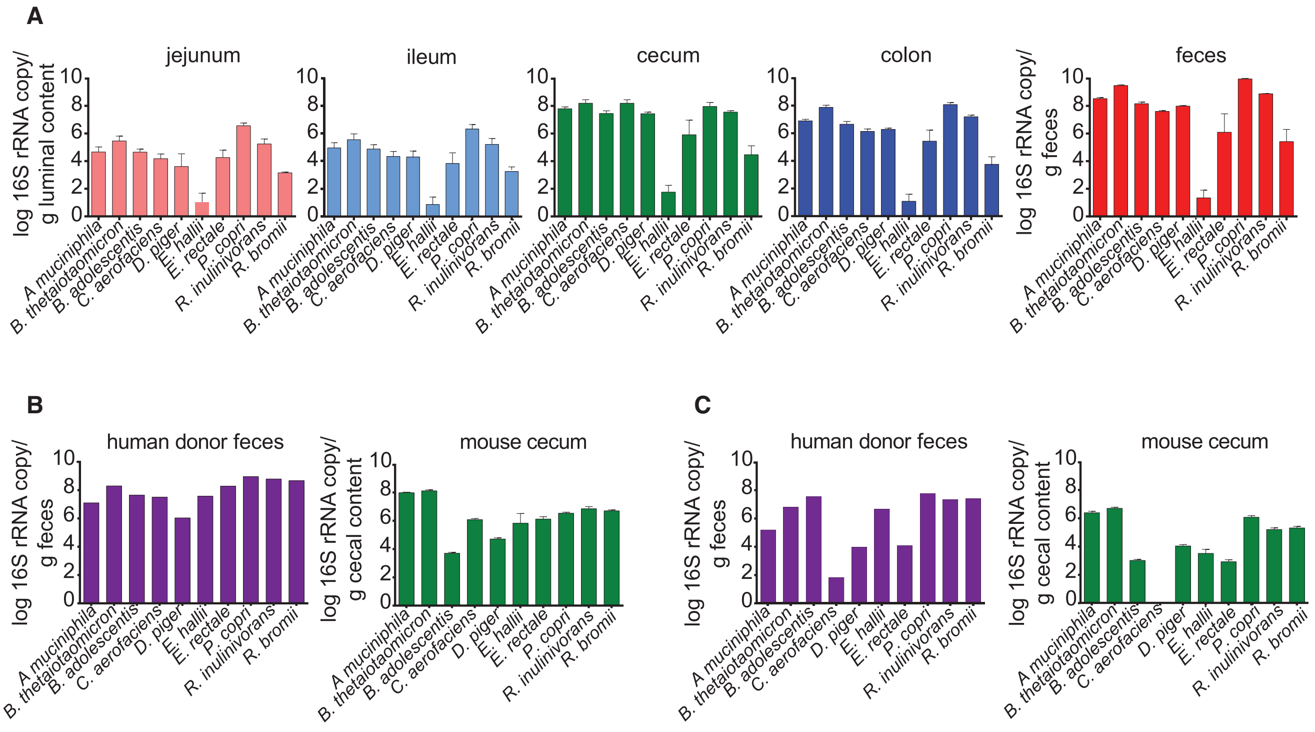

Figure 1. SIM Bacteria Colonize the Mouse Gut

(A) Abundance of each of the SIM bacteria in jejunum, ileum, cecum, colon, and feces of chow-fed SIM male mice (n = 5; mice are from two independent experiments; each sample was analyzed in duplicate in one run and in duplicate PCR runs).

(B) Abundance of each of the SIM bacteria in the feces of a female human donor and in the cecum of chow-fed Swiss Webster female mice (n = 6; each sample was analyzed in duplicate in one run and in duplicate PCR runs) colonized with feces from this donor.

(C) Abundance of each of the SIM bacteria in the feces of a male human donor and in the cecum of chow-fed Swiss Webster male mice (n = 5; each sample was analyzed in duplicate in one run and in duplicate PCR runs) colonized with feces from this donor.

Data are mean ± SEM.

Dietary Changes Modify the Colonization Pattern of the Cecal SIM Community

A major selection criterion of the SIM bacteria was their capacity to metabolize carbohydrates and participate in anaerobic food webs in the gut. We therefore assessed how the abundance of SIM members was affected by changing the mouse diet from chow, which is low in fat (9% by weight) and high in dietary fiber (i.e., plant polysaccharides; 15% by weight), to one of two purified diets with low dietary fiber (primarily cellulose) but different macronutrient compositions. One group of SIM mice received a "Western" high-fat/high-sucrose (HF-HS) diet (fat 20% by weight, sucrose 18% by weight) for 2 weeks; a second group received a zero-fat/high-sucrose (ZF-HS) diet (sucrose 63% by weight) for 2 weeks (Table S1); a third group remained on chow.

The total bacterial load in the cecum of SIM mice was not affected by a change in the diet (Figure 2A). However, compared with SIM mice that remained on chow, SIM mice that switched to a diet low in dietary fiber had reduced cecal abundance of *Prevotella copri* and *Bifidobacterium adolescentis* (with either HF-HS or ZF-HS diet), *Ruminococcus bromii* (with HF-HS diet), and *R. inulinivorans* (with ZF-HS diet) as well as a trend toward reduced abundance of *C. aerofaciens* (with ZF-HS diet; p = 0.052) (Figure 2A). Thus, a reduction in dietary fiber reduced the abundance of these fiber-degrading bacteria. These results are consistent with earlier studies showing that *P. copri*, *B. adolescentis*, *R. inulinivorans*, *R. bromii*, and *C. aerofaciens* have increased abundance when the diet is rich in complex plant polysaccharides (Kovatcheva-Datchary et al., 2015; Ramirez-Farias et al., 2009; Scott et al., 2014; Walker et al., 2011; Vital et al., 2018). Furthermore, a recent study reported that bacteria from the phylogenetic order Bacteroidales (to which *Prevotella* species belong) are lost in mice after several generations of feeding with a Western diet (Sonnenburg et al., 2016). Similarly, a loss of *Prevotella* strains has been reported in the gut microbiome of non-Western individuals after immigration to a Western country (Vangay et al., 2018). In addition, two recent studies in humans showed that short-term dietary changes to reduce complex carbohydrates resulted in rapid reductions in abundance of *Bifidobacterium*, *Ruminococcus*, and *Roseburia*, bacteria that metabolize complex carbohydrates in the gut (David et al., 2014; Mardinoglu et al., 2018).

The cecal abundance of *E. hallii* was higher in SIM mice that switched from chow to an HF-HS or a ZF-HS diet (Figure 2A). In agreement, a dietary intervention study in humans showed that resistant starch (a prominent dietary fiber) reduced the abundance of *E. hallii* (Salonen et al., 2014). Furthermore,

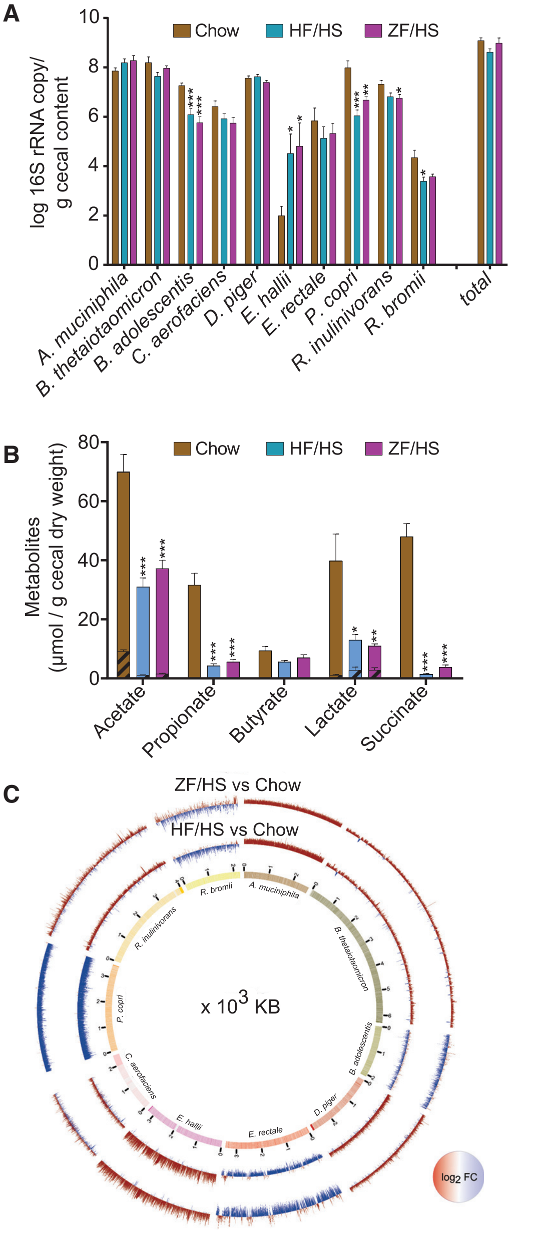

Figure 2. Dietary Changes Affect the Cecal SIM Community

(A) Abundance of each of the SIM bacteria in the cecum of SIM mice that remained on chow or switched to an HF-HS diet or a ZF-HS diet for 2 weeks (n = 8–11; mice are from two independent experiments; each sample was analyzed in duplicate in one run and in duplicate PCR runs).

(B) Concentrations of SCFAs and organic acids in the cecum of GF mice (n = 5 or 6) and SIM mice that remained on chow or switched to an HF-HS diet or a ZF-HS diet for 2 weeks (n = 8–11; mice are from two independent experiments). Metabolite concentrations for GF mice are shown by hashed lines and overlay data for SIM mice. Data are mean ± SEM. *p < 0.05, **p < 0.01, and ***p < 0.001 versus chow (one-way ANOVA).

(C) Positive (red) and negative (blue) fold changes in cecal expression of genes for each of the SIM bacteria in mice that switched to a ZF-HS diet (outer circle) or an HF-HS diet (inner circle) compared with mice that remained on chow for 2 weeks (n = 5; mice are from two independent experiments). Inner circle, genome coverage of generated RNA-seq data.

See also Table S2.

in vitro studies have shown that *E. hallii* can use a broad range of substrates, including glucose, lactose, galactose, glycerol, and amino sugars (Bunesova et al., 2018; Duncan et al., 2004a; Engels et al., 2016; Scott et al., 2014). We observed a non-significant increase in the abundance of *Akkermansia muciniphila* and no change in the abundance of *Bacteroides thetaiotaomicron* or *Eubacterium rectale* in SIM mice that switched from chow to an HF-HS or a ZF-HS diet (Figure 2A), consistent with the fact that these bacteria are not exclusively dependent on dietary fiber and can degrade host mucin or simple sugars from the diet. *A. muciniphila* is an avid user of mucin glycans (Derrien et al., 2004). *B. thetaiotaomicron* is able to digest a broad range of dietary polysaccharides and adapts to different sources of carbohydrates, including mucin glycans, in the absence of dietary fibers (Sonnenburg et al., 2005). *E. rectale* can metabolize different carbohydrates (amylopectin, amylose, arabinoxylan, fructo-oligosaccharide, galacto-oligosaccharide, inulin, xylo-oligosaccharide) (Sheridan et al., 2016) but has also been shown to grow well on glucose (Cockburn et al., 2015; Ze et al., 2012).

The abundance of *D. piger* was also not affected by a change in diet (Figure 2A). The growth and survival of *D. piger* is dependent on the availability of sulfate (Rey et al., 2013), which in the mammalian gut is sourced not only from the diet but also from sulfated glycans in host mucin. Although *D. piger* lacks sulfatase activity, *A. muciniphila* and *B. thetaiotaomicron* are capable of removing sulfate from sulfated glycans when metabolizing host glycans and thus can contribute to the availability of sulfate required by *D. piger* (Rey et al., 2013).

There were no significant differences in individual bacterial species between mice on an HF-HS or a ZF-HS diet (Figure 2A), indicating that a reduction in plant polysaccharides has a greater influence on the abundance of the SIM members than variations in either fat or sucrose.

Dietary Changes Affect the Fermentative Capacity of the Cecal SIM Community

We confirmed the functional capacity of the SIM community to metabolize carbohydrates by showing that levels of SCFAs and organic acids were dramatically higher in cecal samples from SIM mice compared with GF mice on a chow diet (Figure 2B). To investigate the functional response of the SIM microbiota to dietary changes, we also compared SCFA levels in cecal samples from SIM mice that switched to an HF-HS or a ZF-HS diet with those that remained on chow. We showed that a switch to a diet low in plant polysaccharides resulted in reduced cecal concentrations of the SCFAs acetate and propionate as well as of the organic acids lactate and succinate (Figure 2B), in parallel with the reduced abundance of *P. copri*, *B. adolescentis*, *R. bromii*, and *R. inulinivorans* observed in response to this diet change (Figure 2A).

Butyrate was not significantly affected when SIM mice switched to a ZF-HS or an HF-HS diet (Figure 2B), consistent with the unchanged/increased abundance of the known butyrate producers *E. rectale* and *E. hallii* in response to these diets change (Figure 2A). Previous *in vitro* findings have shown that *E. hallii* can produce butyrate from glucose, lactate, acetate, and mucin-derived monosaccharides (Bunesova et al., 2018; Duncan et al., 2004b; Scott et al., 2014; Shetty et al., 2017).

Dietary Changes Modify the Transcriptome of the Cecal SIM Community

To further investigate the functional response of the SIM community to dietary changes, we performed RNA sequencing on cecal microbiota from the SIM mice that remained on chow or switched to an HF-HS or a ZF-HS diet for 2 weeks. We obtained an average depth of 15 million paired-end reads per sample, which was sufficient to cover the genomes of each SIM bacterium (Figure 2C). We identified differences (adjusted $p < 0.05$) in 5,765 and 3,134 genes in cecal samples from the mice that switched to an HF-HS and a ZF-HS diet, respectively, compared with SIM mice that remained on chow (Table S2). By contrast, only 166 genes were significantly differently expressed when comparing samples from the mice on an HF-HS versus a ZF-HS diet (Table S2). These results demonstrate that gene expression in the SIM cecal microbiota is influenced to a greater extent by a reduction in plant polysaccharides than variations in either fat or sucrose.

We compared differences in gene expression at the species level in SIM mice fed an HF-HS or a ZF-HS diet with SIM mice fed chow and observed that (1) *E. hallii*, *A. muciniphila*, *B. thetaiotaomicron*, and *D. piger* had mostly upregulated genes, consistent with increased or no change in abundance of these species in the absence of plant polysaccharides, and (2) *P. copri* had exclusively downregulated genes, consistent with its reduced abundance in the absence of fiber (Figure 2C). In addition, *R. inulinivorans*, *R. bromii*, *B. adolescentis*, *E. rectale*, and *C. aerofaciens* had both upregulated and downregulated genes (Figure 2C), consistent with the fact that these SIM bacteria are capable of engaging in different trophic interactions (e.g., by competition and cooperation) to meet their nutrient requirements. These species can be primary degraders of dietary carbohydrates (Flint et al., 2012; Pokusaeva et al., 2011; Scott et al., 2011; Sheridan et al., 2016; Ze et al., 2012) and are also involved in metabolic cross-feeding interactions to maintain gut homeostasis (Belenguer et al., 2006; Crost et al., 2018; Flint et al., 2015; Marquet et al., 2009).

To explore functional features, we next used KEGG orthology (KO) and the protein domain database (Pfam) to annotate genes that were significantly altered in cecal samples from mice fed HF-HS or ZF-HS compared with mice fed chow for each member of the SIM microbiota (Table S2). A switch to reduced plant polysaccharides in the diet resulted in (1) decreases in KOs associated with the fermentation of plant polysaccharides (e.g., alpha-amylase, beta-glucosidase, endoglucanase, and xylan 1,4-beta-xylosidase), specifically in *P. copri*, *E. rectale*, and *B. adolescentis*; (2) increases in KOs associated with the conversion of simple sugars (such as ribose, ribulose, xylulose, fructose, mannose, sucrose, and glucose), amino sugars, pyruvate, and SCFAs (mostly butyrate and propionate), particularly in *B. thetaiotaomicron*, *A. muciniphila*, *E. hallii*, and *R. inulinivorans*; and (3) increases in KOs associated with the metabolism of mucin, particularly in *A. muciniphila* related to the degradation of mucin O-glycans and in *E. hallii* related to the metabolism of mucin monosaccharides (primarily galactose, glucose, and N-acetylglucosamine) (Table S2). For most of the genes that were significantly altered in SIM mice that switched to reduced plant polysaccharides in the diet, we obtained similar annotations with both Pfam domains and KOs (Table S2). The most frequent Pfam annotations in genes that were significantly altered included those that were related to amino acid metabolism and to the transport of degraded carbohydrates (Table S2).

Dietary Changes Affect Carbohydrate-Active Enzyme Genes in the Cecal SIM Community

To investigate how dietary changes affect the contribution of the individual bacteria to the conversion of dietary carbohydrates, we next analyzed changes in SIM transcripts encoding carbohydrate-active enzymes (CAZymes) in cecal samples from mice fed an HF-HS or a ZF-HS diet compared with mice fed chow using the meta server dbCAN2, which integrates three tools for CAZyme genome annotation (Zhang et al., 2018). We observed similar results with all three tools (Table S3). In total, we identified diet-induced alterations in genes encoding 192 unique CAZymes belonging to 24 different families, including (1) enzymes involved in the assembly of carbohydrates such as glycosyl transferases (Lairson et al., 2008); (2) enzymes involved in carbohydrate breakdown including auxiliary activity enzymes, carbohydrate esterases, glycoside hydrolases, and polysaccharide lyases; and (3) accessory modules such as carbohydrate-binding modules (El Kaoutari et al., 2013) (Table S3).

A subset of these CAZymes showed the same pattern of expression changes in SIM mice that switched to either an HF-HS or a ZF-HS diet compared with SIM mice that remained on chow (Figure 3). For example, we observed reduced gene expression of *P. copri*-affiliated CAZymes potentially involved in the conversion of plant polysaccharides (including cellulose, hemicellulose, beta-glucans, beta-mannan, pectin, and starch) in mice that switched to either of the fiber-reduced diets (Figure 3), in parallel with the reduced abundance of *P. copri* in these mice (Figure 2A) and consistent with studies showing that *Prevotella* abundance is associated with fiber intake in humans (David et al., 2014; Wu et al., 2011). In addition, we observed increased gene expression of *A. muciniphila*-affiliated CAZymes potentially involved in the metabolism of host glycans and mucin in mice that switched to either of the fiber-reduced diets (Figure 3). A recent study in mice colonized with a synthetic human gut microbiota reported enhanced mucin degradation in response to a fiber-deprived diet (Desai et al., 2016). In this earlier study, *A. muciniphila* and *Bacteroides caccae* but not *B. thetaiotaomicron* contributed to the degradation of host glycans and mucin. However, it is likely that *B. thetaiotaomicron* is outcompeted by *B. caccae* if both are present; *B. caccae* had a higher colonization rate in mice colonized with selected human bacteria and fed a fiber-free diet (Desai et al., 2016) and has earlier been shown to have a higher growth rate on mucin and host glycans *in vitro* (Desai et al., 2016; McNulty et al., 2013).

A number of the diet-induced changes in CAZyme gene expression were specific to the dietary change (Figure 3). For example, in mice that switched to an HF-HS diet, we observed increased gene expression of CAZymes associated with conversion of plant polysaccharides, degradation of mucin, and host glycans, and synthesis of lipopolysaccharide and affiliated with *E. hallii*, *A. muciniphila*, *R. inulinivorans*, and *B. adolescentis*

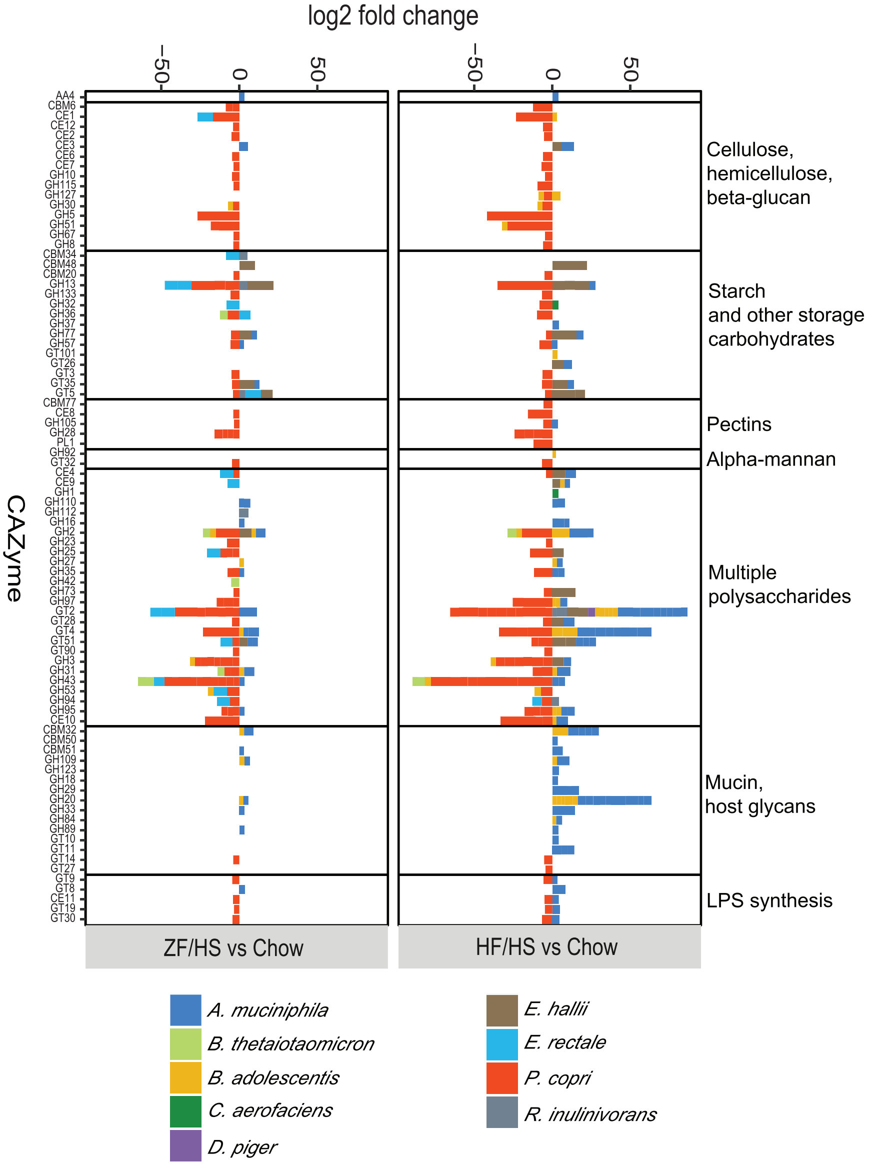

Figure 3. Dietary Changes Affect CAZyme Expression in the Cecal SIM Community

Log fold changes in transcripts encoding CAZymes in the metatranscriptomics data of cecal samples from SIM mice fed HF-HS or ZF-HS compared with SIM mice fed chow (n = 5 mice/group; mice are from two independent experiments). Only the CAZyme families with adjusted p values < 0.05 are shown as averages. We classified CAZyme families on the basis of respective substrates; GH5 can potentially convert beta-mannan in addition to cellulose, hemicellulose, and beta-glucans. AA, auxiliary activities; CBM, carbohydrate-binding module; CE, carbohydrate esterase; GH, glycoside hydrolase; GT, glycosyl transferase; PL, polysaccharide lyase.

See also Table S3.

(Figure 3). In contrast, only a few transcripts were increased specifically in mice that switched to a ZF-HS diet (Figure 3); these transcripts mostly belong to glycosyl transferase families, which are involved in carbohydrate assembly rather than degradation (Lairson et al., 2008). Furthermore, we observed reduced gene expression of *E. rectale*-affiliated CAZymes associated with conversion of plant polysaccharides mainly in SIM mice that switched to a ZF-HS diet (glycoside hydrolase 94 was the only *E. rectale*-affiliated CAZyme that showed reduced gene expression when SIM mice switched to either an HF-HS or a ZF-HS diet) (Figure 3).

Together these findings show the flexibility of the SIM community to efficiently metabolize the carbohydrates from the diet. They also indicate that the diet-induced reduction in the fermentative capacity of the SIM community is not a result of high levels of fat in the diet but rather is caused by the reduction in carbohydrates that can be metabolized by the bacteria.

Dietary Changes Affect SIM-Produced Metabolites in the Portal Vein

To investigate the effect of microbe-diet interactions on circulating metabolites, we performed metabolomics on plasma samples collected from the portal vein of the SIM mice that switched to an HF-HS or a ZF-HS diet for 2 weeks and from those that remained on chow. To discriminate between host and microbially derived plasma metabolites, we also analyzed plasma samples from GF mice on the three different diets. In total, 548 metabolites were measured (Table S4); of these, metabolites whose levels were significantly altered by the microbiota (75) or by diet change (89) are shown in Figures 4A and 4B, respectively. We identified 17 metabolites that were both microbially regulated and modulated by dietary changes in plasma samples from SIM mice (shown in red text in Figures 4A and 4B). These metabolites were mostly linked to lipid and amino acid metabolism.

Specifically, the 17 microbially regulated metabolites that were affected by reducing the consumption of dietary fiber were (1) the unsaturated fatty acids oleate and 10-nanodecenoate (increased in SIM mice on HF-HS or ZF-HS versus chow); (2) the acylcarnitines 2-methylbutyrylcarnitine, 2-methylbutyrylglycine, isobutyrylcarnitine, propionylcarnitine, and propionylglycine; the purine metabolite xanthosine; succinate; and ferulic acid 4-sulfate (decreased in SIM mice on HF-HS or ZF-HS versus chow); and (3) kynurenate, anthranilate, and xanthurenate, key metabolites in the kynurenine pathway of

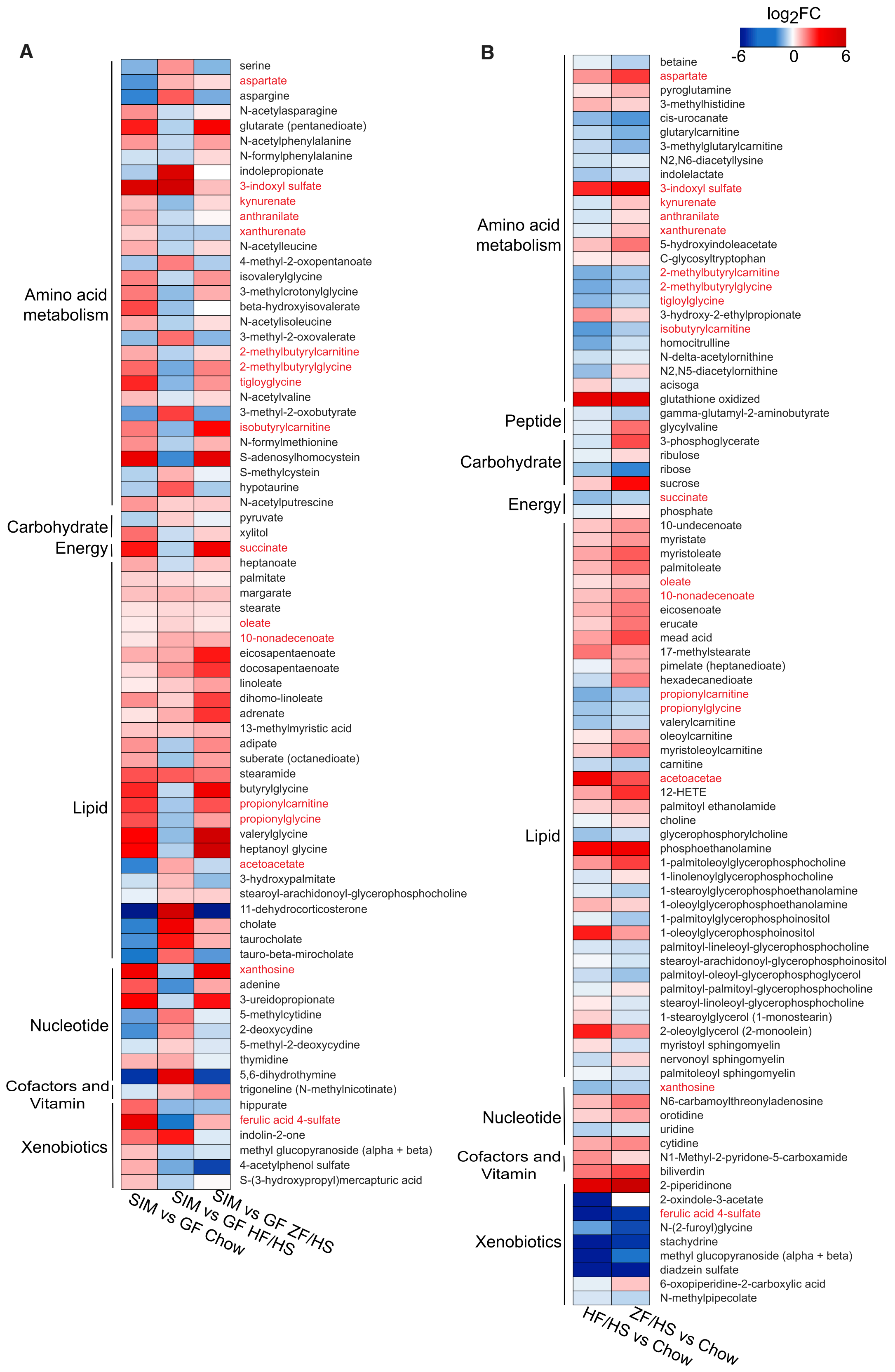

(legend on next page)

tryptophan catabolism (decreased in SIM mice on HF-HS versus chow and increased in SIM mice on ZF-HS versus chow) (Figure 4B; Table S4).

The reduction of succinate in the portal vein of SIM mice on an HF-HS or a ZF-HS diet versus chow was consistent with reduced cecal levels of succinate (Figure 2B). *P. copri* is an efficient succinate producer (Franke and Deppenmeier, 2018; Kovatcheva-Datchary et al., 2015), and we have previously demonstrated that succinate may improve metabolism by stimulating intestinal gluconeogenesis (De Vadder et al., 2016). Ferulic acid 4-sulfate, which was also reduced in the portal vein of SIM mice on an HF-HS or a ZF-HS diet versus chow, is obtained after conjugation of ferulic acid during its uptake in the intestinal epithelium and passage through the liver (Bourne and Rice-Evans, 1998). The beneficial effect of ferulic acid and ferulic acid 4-sulfate in diabetes, cardiovascular diseases, improved lipid metabolism, and blood pressure lowering has been well recognized (Alam et al., 2016; Van Rymenant et al., 2017; Vinayagam et al., 2016). Thus, dietary fiber-induced increases in succinate and ferulic acid 4-sulfate may be beneficial for host metabolism.

To investigate if we could identify links between any of these 17 portal vein metabolites (which were both microbially regulated and modulated by dietary changes) and SIM-regulated genes, we searched for SIM- and diet-regulated operons in the metatranscriptome and investigated whether their resulting genes could contribute to SIM- and diet-induced regulation of the portal vein metabolites. We identified one operon (of *E. rectale* consisting of EUBREC_1041 and EUBREC_1042) that was significantly reduced in the cecal transcriptome of SIM mice on a ZF-HS diet versus chow (Table S2). EUBREC_1041 encodes acyl-CoA thioesterase (Mahowald et al., 2009) and EUBREC_1042 encodes arabinofuranosidase (also termed xylan 1,4-beta-xylosidase), which can release ferulic acid from dietary fibers (Duncan et al., 2016; Nishizawa et al., 1998). We next searched the CAZyme dataset for enzyme families that could contribute to microbiota activity associated with ferulic acid release. We observed that transcripts of the carbohydrate esterase family CE1, which includes ferulic acid esterase activity (Dodd and Cann, 2009), were decreased in cecal samples from mice on a ZF-HS diet versus chow, with a major contribution of *E. rectale* (Figure 3). Thus, we propose that *E. rectale*, which is abundant in the human microbiome, may contribute to ferulic acid release from diets rich in plant polysaccharides in the mammalian gut.

SIM Affects Host Metabolism in a Diet-Specific Fashion

To investigate whether the SIM system also could be used to study microbe-diet effects on host metabolism, we compared body weight, adiposity, steatosis, and glucose metabolism in SIM versus GF mice on each of the three diets.

In mice on chow, we observed that SIM was sufficient to increase body weight and epididymal adipose weight, induce hepatic steatosis, and increase blood glucose levels (Figures 5A–5E). Thus, SIM has effects on host metabolism similar to those seen in mice colonized with a normal mouse microbiota (Bäckhed et al., 2004; Caesar et al., 2012) and may induce adiposity, resulting in impaired glucose tolerance by enhancing energy harvest (i.e., increased SCFA production from dietary fibers) in the host.

In mice on an HF-HS diet, we again demonstrated that SIM increased blood glucose levels (Figure 5E). However, this response was not paralleled by an increase in body weight (Figure 5A), in contrast to our earlier study comparing HF-HS diet-fed conventionally raised and GF mice (Bäckhed et al., 2007). This discrepancy may be explained by the fact that the present diet intervention was shorter than in the previous study (2 versus 8 weeks) and that another mouse strain was used (Swiss Webster versus C57BL/6). However, we observed increased adiposity and steatosis in SIM versus GF mice on an HF-HS diet (Figures 5B–5D), and both of these responses are known to be associated with impaired glucose tolerance (Cani et al., 2007).

In mice on the most extreme diet, ZF-HS, we observed that SIM had its main effect on hepatic steatosis, resulting in a 2.8 $\pm$ 0.5 fold increase in liver fat compared with GF mice on the same diet (Figure 5D). SIM promoted a small increase in body weight and epididymal white adipose weight (Figures 5A and 5C), without affecting overall adiposity determined by magnetic resonance imaging (Figure 5B) in mice on a ZF-HS diet. Surprisingly, we did not observe significant changes in blood glucose levels in SIM mice compared with GF mice on the ZF-HS diet (Figure 5E), despite the difference in hepatic steatosis. One explanation can be that the ZF-HS diet has direct effects on glucose metabolism in GF mice, independently of steatosis, as the glucose excursion curve was elevated in GF mice on this diet compared with GF mice fed chow or an HF-HS diet (Figure 5E).

Prospectus

Studies of the gut microbiota are complex and often limited by a lack of sequenced strains and poor gene annotations. To overcome these limitations, we colonized GF mice with a set of relatively well-characterized bacteria that represent the main phyla in the human gut and used the model to investigate how SIM-diet interactions affect the transcriptome, metabolome, and host metabolism. The SIM community has many of the features of the complete microbiota in modulating host metabolism in a diet-dependent fashion and, as such, the SIM model may facilitate our understanding of how the microbes interact with macronutrients to affect host metabolism. Because we use bacterial strains isolated from humans, our findings may be extrapolated to humans and potentially provide guidance for human

Figure 4. Dietary Changes and Microbiota Affect Plasma Metabolites

(A) Heatmap showing statistically significant fold changes in concentrations of metabolites in portal vein plasma from SIM mice (n = 5–7; mice are from two independent experiments) versus GF mice (n = 6–8) on chow, HF-HS, and ZF-HS diets.

(B) Heatmap showing statistically significant fold changes in concentrations of metabolites in portal vein plasma from SIM mice on HF-HS (n = 7; mice are from two independent experiments) or ZF-HS (n = 5; mice are from two independent experiments) diet versus SIM mice on chow (n = 7; mice are from two independent experiments) (false discovery rate [FDR], $q < 0.1$). Metabolites significantly regulated in both datasets (A and B) are listed in red.

See also Table S4.

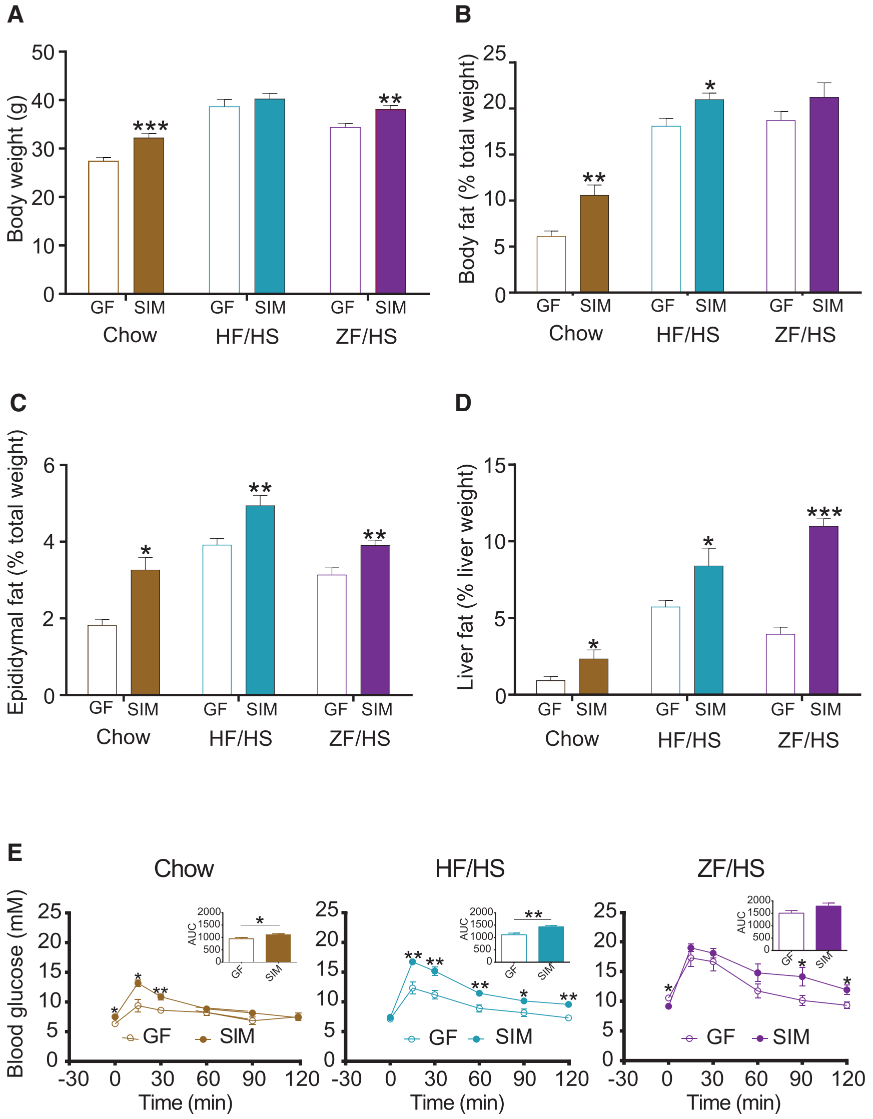

Figure 5. Metabolic Phenotypes of the SIM Mice in Response to Diet

(A–E) Body weight (A), body fat (B), epididymal fat (C), liver fat (D), and blood glucose (E) of SIM mice (n = 5) compared with GF mice (n = 5 or 6) on chow, HF-HS, and ZF-HS diets. Data are mean ± SEM. *p < 0.05, **p < 0.01, and ***p < 0.001 (Student's t test).

intervention studies. However, further experiments that consider the complexity of human diets and the variety of dietary fibers are required to determine how well data from our simplified system can be translated into human physiology.

STAR★METHODS

Detailed methods are provided in the online version of this paper and include the following:

- KEY RESOURCES TABLE
- CONTACT FOR REAGENT AND RESOURCE SHARING
- EXPERIMENTAL MODEL AND SUBJECT DETAILS
  - Colonization of mice with the SIM bacteria
  - Colonization of mice with human feces
  - Dietary experiments with SIM mice
  - Bacteria strains
- METHOD DETAILS
  - Body composition and glucose tolerance test
  - Genomic DNA extraction and 16S rRNA quantitative PCR
  - Quantification of SCFAs and organic acids
  - Microbial RNA extraction
  - Metatranscriptome analysis of the SIM community
  - Metabolomic analysis of plasma from SIM mice
- QUANTIFICATION AND STATISTICAL ANALYSIS
- DATA AND SOFTWARE AVAILABILITY

SUPPLEMENTAL INFORMATION

Supplemental Information can be found with this article online at https://doi.org/10.1016/j.celrep.2019.02.090.

ACKNOWLEDGMENTS

We are grateful to Carina Arvidsson, Sara Nordin-Larsson, Ulrica Enqvist, Caroline Wennberg, and Zakarias Gulic for excellent animal husbandry and

the genomics core facility at Sahlgrenska Academy for performing microbiota RNA sequencing (RNA-seq). We thank Dr. Karen Scott, Dr. Sylvia Duncan, and Jenny Martin of the Rowett Institute in Nutrition and Health (Aberdeen, UK) for fruitful discussions during the design of the SIM community and for providing *E. hallii* L2-7 (DSM 17630), *E. rectale* A1-86 (DSM 17629), *B. adolescentis* L2-32, *C. aerofaciens* (DSM 3979), *D. piger* (DSM 749), *R. inulinivorans* A2-194 (DSM 16841), and *R. bromii* L2-63. We thank Professor Willem de Vos and Dr. Clara Berzel of Wageningen University (the Netherlands) for providing us with *A. muciniphila* (DSM 22959). This study was supported by a DuPont Young Professor Award, the Swedish Research Council, IngaBritt and Arne Lundberg's Foundation, JPI (A healthy diet for a healthy life; 2017-01996_3), the Knut and Alice Wallenberg Foundation, the Novo Nordisk Foundation (NNF13OC008163), the Leducq Foundation (17CVD01), and a grant from the Swedish state under the agreement between the Swedish government and the county councils, the ALF-agreement (ALFGBG-718101). F.B. is a recipient of a European Research Council (ERC) Consolidator Grant (615362; METABASE) and is the Torsten Söderberg Professor in Medicine. P.K.-D. is a recipient of fellowships from the CAS President's International Fellowship Initiative (PIFI; project 2018PB0028) and DICP Outstanding Postdoctoral Foundation (grant 2017YB05). S.S. is a recipient of a fellowship from the Engineering and Physical Sciences Research Council (EPSRC) (project EP/S001301/1).

AUTHOR CONTRIBUTIONS

P.K.-D. and F.B. conceived and designed the project. P.K.-D., A.H., and A.W. collected samples. P.K.-D., S.S, and A.W. performed experiments. I.N. performed statistical and normalization of transcription data. S.L. and S.S. performed the functional analysis of data. P.K.-D., S.S., I.N., A.W., R.P., J.N., and F.B. analyzed and interpreted the data. P.K.-D., S.S., R.P., and F.B. wrote the paper. All authors commented on the manuscript.

DECLARATION OF INTERESTS

J.N. and F.B. are founders and shareholders of Metabogen AB.

Received: November 2, 2018
Revised: January 22, 2019
Accepted: February 21, 2019
Published: March 26, 2019

REFERENCES

Alam, M.A., Subhan, N., Hossain, H., Hossain, M., Reza, H.M., Rahman, M.M., and Ullah, M.O. (2016). Hydroxycinnamic acid derivatives: a potential class of natural compounds for the management of lipid metabolism and obesity. Nutr. Metab. (Lond.) *13*, 27.

Anders, S., Pyl, P.T., and Huber, W. (2015). HTSeq—a Python framework to work with high-throughput sequencing data. Bioinformatics *31*, 166–169.

Arumugam, M., Raes, J., Pelletier, E., Le Paslier, D., Yamada, T., Mende, D.R., Fernandes, G.R., Tap, J., Bruls, T., Batto, J.-M., et al.; MetaHIT Consortium (2011). Enterotypes of the human gut microbiome. Nature *473*, 174–180.

Bäckhed, F., Ding, H., Wang, T., Hooper, L.V., Koh, G.Y., Nagy, A., Semenkovich, C.F., and Gordon, J.I. (2004). The gut microbiota as an environmental factor that regulates fat storage. Proc. Natl. Acad. Sci. U S A *101*, 15718–15723.

Bäckhed, F., Manchester, J.K., Semenkovich, C.F., and Gordon, J.I. (2007). Mechanisms underlying the resistance to diet-induced obesity in germ-free mice. Proc. Natl. Acad. Sci. U S A *104*, 979–984.

Becker, N., Kunath, J., Loh, G., and Blaut, M. (2011). Human intestinal microbiota: characterization of a simplified and stable gnotobiotic rat model. Gut Microbes *2*, 25–33.

Belenguer, A., Duncan, S.H., Calder, A.G., Holtrop, G., Louis, P., Lobley, G.E., and Flint, H.J. (2006). Two routes of metabolic cross-feeding between *Bifidobacterium adolescentis* and butyrate-producing anaerobes from the human gut. Appl. Environ. Microbiol. *72*, 3593–3599.

Bourne, L.C., and Rice-Evans, C. (1998). Bioavailability of ferulic acid. Biochem. Biophys. Res. Commun. *253*, 222–227.

Bunesova, V., Lacroix, C., and Schwab, C. (2018). Mucin cross-feeding of infant *Bifidobacteria* and *Eubacterium hallii*. Microb. Ecol. *75*, 228–238.

Caesar, R., Reigstad, C.S., Bäckhed, H.K., Reinhardt, C., Ketonen, M., Lundén, G.Ö., Cani, P.D., and Bäckhed, F. (2012). Gut-derived lipopolysaccharide augments adipose macrophage accumulation but is not essential for impaired glucose or insulin tolerance in mice. Gut *61*, 1701–1707.

Cani, P.D., Hoste, S., Guiot, Y., and Delzenne, N.M. (2007). Dietary non-digestible carbohydrates promote L-cell differentiation in the proximal colon of rats. Br. J. Nutr. *98*, 32–37.

Cockburn, D.W., Orlovsky, N.I., Foley, M.H., Kwiatkowski, K.J., Bahr, C.M., Maynard, M., Demeler, B., and Koropatkin, N.M. (2015). Molecular details of a starch utilization pathway in the human gut symbiont *Eubacterium rectale*. Mol. Microbiol. *95*, 209–230.

Collado, M.C., Derrien, M., Isolauri, E., de Vos, W.M., and Salminen, S. (2007). Intestinal integrity and *Akkermansia muciniphila*, a mucin-degrading member of the intestinal microbiota present in infants, adults, and the elderly. Appl. Environ. Microbiol. *73*, 7767–7770.

Crost, E.H., Le Gall, G., Laverde-Gomez, J.A., Mukhopadhya, I., Flint, H.J., and Juge, N. (2018). Mechanistic insights into the cross-feeding of *Ruminococcus gnavus* and *Ruminococcus bromii* on host and dietary carbohydrates. Front. Microbiol. *9*, 2558.

Cummings, J.H., and Macfarlane, G.T. (1991). The control and consequences of bacterial fermentation in the human colon. J. Appl. Bacteriol. *70*, 443–459.

David, L.A., Maurice, C.F., Carmody, R.N., Gootenberg, D.B., Button, J.E., Wolfe, B.E., Ling, A.V., Devlin, A.S., Varma, Y., Fischbach, M.A., et al. (2014). Diet rapidly and reproducibly alters the human gut microbiome. Nature *505*, 559–563.

De Vadder, F., Kovatcheva-Datchary, P., Zitoun, C., Duchampt, A., Bäckhed, F., and Mithieux, G. (2016). Microbiota-produced succinate improves glucose homeostasis via intestinal gluconeogenesis. Cell Metab. *24*, 151–157.

Derrien, M., Vaughan, E.E., Plugge, C.M., and de Vos, W.M. (2004). *Akkermansia muciniphila* gen. nov., sp. nov., a human intestinal mucin-degrading bacterium. Int. J. Syst. Evol. Microbiol. *54*, 1469–1476.

Desai, M.S., Seekatz, A.M., Koropatkin, N.M., Kamada, N., Hickey, C.A., Wolter, M., Pudlo, N.A., Kitamoto, S., Terrapon, N., Muller, A., et al. (2016). A dietary fiber-deprived gut microbiota degrades the colonic mucus barrier and enhances pathogen susceptibility. Cell *167*, 1339–1353.e21.

Dethlefsen, L., McFall-Ngai, M., and Relman, D.A. (2007). An ecological and evolutionary perspective on human-microbe mutualism and disease. Nature *449*, 811–818.

Dobin, A., Davis, C.A., Schlesinger, F., Drenkow, J., Zaleski, C., Jha, S., Batut, P., Chaisson, M., and Gingeras, T.R. (2013). STAR: ultrafast universal RNA-seq aligner. Bioinformatics *29*, 15–21.

Dodd, D., and Cann, I.K.O. (2009). Enzymatic deconstruction of xylan for biofuel production. Glob. Change Biol. Bioenergy *1*, 2–17.

Duncan, S.H., and Flint, H.J. (2008). Proposal of a neotype strain (A1-86) for *Eubacterium rectale*. Request for an opinion. Int. J. Syst. Evol. Microbiol. *58*, 1735–1736.

Duncan, S.H., Holtrop, G., Lobley, G.E., Calder, A.G., Stewart, C.S., and Flint, H.J. (2004a). Contribution of acetate to butyrate formation by human faecal bacteria. Br. J. Nutr. *91*, 915–923.

Duncan, S.H., Louis, P., and Flint, H.J. (2004b). Lactate-utilizing bacteria, isolated from human feces, that produce butyrate as a major fermentation product. Appl. Environ. Microbiol. *70*, 5810–5817.

Duncan, S.H., Aminov, R.I., Scott, K.P., Louis, P., Stanton, T.B., and Flint, H.J. (2006). Proposal of *Roseburia faecis* sp. nov., *Roseburia hominis* sp. nov. and *Roseburia inulinivorans* sp. nov., based on isolates from human faeces. Int. J. Syst. Evol. Microbiol. *56*, 2437–2441.

Duncan, S.H., Lobley, G.E., Holtrop, G., Ince, J., Johnstone, A.M., Louis, P., and Flint, H.J. (2008). Human colonic microbiota associated with diet, obesity and weight loss. Int. J. Obes. *32*, 1720–1724.

Duncan, S.H., Russell, W.R., Quartieri, A., Rossi, M., Parkhill, J., Walker, A.W., and Flint, H.J. (2016). Wheat bran promotes enrichment within the human colonic microbiota of butyrate-producing bacteria that release ferulic acid. Environ. Microbiol. *18*, 2214–2225.

Duranti, S., Milani, C., Lugli, G.A., Mancabelli, L., Turroni, F., Ferrario, C., Mangifesta, M., Viappiani, A., Sánchez, B., Margolles, A., et al. (2016). Evaluation of genetic diversity among strains of the human gut commensal *Bifidobacterium adolescentis*. Sci. Rep. *6*, 23971.

Eddy, S.R. (1998). Profile hidden Markov models. Bioinformatics *14*, 755–763.

Egert, M., de Graaf, A.A., Maathuis, A., de Waard, P., Plugge, C.M., Smidt, H., Deutz, N.E.P., Dijkema, C., de Vos, W.M., and Venema, K. (2007). Identification of glucose-fermenting bacteria present in an *in vitro* model of the human intestine by RNA-stable isotope probing. FEMS Microbiol. Ecol. *60*, 126–135.

El Kaoutari, A., Armougom, F., Gordon, J.I., Raoult, D., and Henrissat, B. (2013). The abundance and variety of carbohydrate-active enzymes in the human gut microbiota. Nat. Rev. Microbiol. *11*, 497–504.

Engels, C., Ruscheweyh, H.-J., Beerenwinkel, N., Lacroix, C., and Schwab, C. (2016). The common gut microbe *Eubacterium hallii* also contributes to intestinal propionate formation. Front. Microbiol. *7*, 713.

Fischbach, M.A., and Sonnenburg, J.L. (2011). Eating for two: how metabolism establishes interspecies interactions in the gut. Cell Host Microbe *10*, 336–347.

Fite, A., Macfarlane, G.T., Cummings, J.H., Hopkins, M.J., Kong, S.C., Furrie, E., and Macfarlane, S. (2004). Identification and quantitation of mucosal and faecal desulfovibrios using real time polymerase chain reaction. Gut *53*, 523–529.

Flint, H.J., Scott, K.P., Duncan, S.H., Louis, P., and Forano, E. (2012). Microbial degradation of complex carbohydrates in the gut. Gut Microbes *3*, 289–306.

Flint, H.J., Duncan, S.H., Scott, K.P., and Louis, P. (2015). Links between diet, gut microbiota composition and gut metabolism. Proc. Nutr. Soc. *74*, 13–22.

Franke, T., and Deppenmeier, U. (2018). Physiology and central carbon metabolism of the gut bacterium *Prevotella copri*. Mol. Microbiol. *109*, 528–540.

Hayashi, H., Shibata, K., Sakamoto, M., Tomita, S., and Benno, Y. (2007). *Prevotella copri* sp. nov. and *Prevotella stercorea* sp. nov., isolated from human faeces. Int. J. Syst. Evol. Microbiol. *57*, 941–946.

Kageyama, A., and Benno, Y. (2000). Emendation of genus *Collinsella* and proposal of *Collinsella stercoris* sp. nov. and *Collinsella intestinalis* sp. nov. Int. J. Syst. Evol. Microbiol. *50*, 1767–1774.

Kageyama, A., Sakamoto, M., and Benno, Y. (2000). Rapid identification and quantification of *Collinsella aerofaciens* using PCR. FEMS Microbiol. Lett. *183*, 43–47.

Koh, A., De Vadder, F., Kovatcheva-Datchary, P., and Bäckhed, F. (2016). From dietary fiber to host physiology: short-chain fatty acids as key bacterial metabolites. Cell *165*, 1332–1345.

Kovatcheva-Datchary, P., Nilsson, A., Akrami, R., Lee, Y.S., De Vadder, F., Arora, T., Hallen, A., Martens, E., Björck, I., and Bäckhed, F. (2015). Dietary fiber-induced improvement in glucose metabolism is associated with increased abundance of *Prevotella*. Cell Metab. *22*, 971–982.

Krzywinski, M., Schein, J., Birol, I., Connors, J., Gascoyne, R., Horsman, D., Jones, S.J., and Marra, M.A. (2009). Circos: an information aesthetic for comparative genomics. Genome Res. *19*, 1639–1645.

Lairson, L.L., Henrissat, B., Davies, G.J., and Withers, S.G. (2008). Glycosyltransferases: structures, functions, and mechanisms. Annu. Rev. Biochem. *77*, 521–555.

Li, H., Handsaker, B., Wysoker, A., Fennell, T., Ruan, J., Homer, N., Marth, G., Abecasis, G., and Durbin, R.; 1000 Genome Project Data Processing Subgroup (2009). The Sequence Alignment/Map format and SAMtools. Bioinformatics *25*, 2078–2079.

Macfarlane, G.T., and Macfarlane, S. (2012). Bacteria, colonic fermentation, and gastrointestinal health. J. AOAC Int. *95*, 50–60.

Mahowald, M.A., Rey, F.E., Seedorf, H., Turnbaugh, P.J., Fulton, R.S., Wollam, A., Shah, N., Wang, C., Magrini, V., Wilson, R.K., et al. (2009). Characterizing a model human gut microbiota composed of members of its two dominant bacterial phyla. Proc. Natl. Acad. Sci. U S A *106*, 5859–5864.

Makki, K., Deehan, E.C., Walter, J., and Bäckhed, F. (2018). The impact of dietary fiber on gut microbiota in host health and disease. Cell Host Microbe *23*, 705–715.

Mardinoglu, A., Wu, H., Björnson, E., Zhang, C., Hakkarainen, A., Räsänen, S.M., Lee, S., Mancina, R.M., Bergentall, M., Pietiläinen, K.H., et al. (2018). An integrated understanding of the rapid metabolic benefits of a carbohydrate-restricted diet on hepatic steatosis in humans. Cell Metab. *27*, 559–571.e5.

Marquet, P., Duncan, S.H., Chassard, C., Bernalier-Donadille, A., and Flint, H.J. (2009). Lactate has the potential to promote hydrogen sulphide formation in the human colon. FEMS Microbiol. Lett. *299*, 128–134.

Martens, E.C., Chiang, H.C., and Gordon, J.I. (2008). Mucosal glycan foraging enhances fitness and transmission of a saccharolytic human gut bacterial symbiont. Cell Host Microbe *4*, 447–457.

Matsuki, T., Watanabe, K., Fujimoto, J., Miyamoto, Y., Takada, T., Matsumoto, K., Oyaizu, H., and Tanaka, R. (2002). Development of 16S rRNA-gene-targeted group-specific primers for the detection and identification of predominant bacteria in human feces. Appl. Environ. Microbiol. *68*, 5445–5451.

McNulty, N.P., Wu, M., Erickson, A.R., Pan, C., Erickson, B.K., Martens, E.C., Pudlo, N.A., Muegge, B.D., Henrissat, B., Hettich, R.L., and Gordon, J.I. (2013). Effects of diet on resource utilization by a model human gut microbiota containing *Bacteroides cellulosilyticus* WH2, a symbiont with an extensive glycobiome. PLoS Biol. *11*, e1001637.

Miyazaki, K., Martin, J.C., Marinsek-Logar, R., and Flint, H.J. (1997). Degradation and utilization of xylans by the rumen anaerobe *Prevotella bryantii* (formerly *P. ruminicola* subsp. *brevis*) B(1)4. Anaerobe *3*, 373–381.

Murphy, N.R., and Hellwig, R.J. (1996). Improved nucleic acid organic extraction through use of a unique gel barrier material. Biotechniques *21*, 934–936, 938–939.

Nishizawa, C., Ohta, T., Egashira, Y., and Sanada, H. (1998). Ferulic acid esterase activities of typical intestinal bacteria. Food Sci. Technol. Intl. Tokyo *4*, 94–97.

Pokusaeva, K., Fitzgerald, G.F., and van Sinderen, D. (2011). Carbohydrate metabolism in *Bifidobacteria*. Genes Nutr. *6*, 285–306.

Qin, J., Li, R., Raes, J., Arumugam, M., Burgdorf, K.S., Manichanh, C., Nielsen, T., Pons, N., Levenez, F., Yamada, T., et al.; MetaHIT Consortium (2010). A human gut microbial gene catalogue established by metagenomic sequencing. Nature *464*, 59–65.

Ramirez-Farias, C., Slezak, K., Fuller, Z., Duncan, A., Holtrop, G., and Louis, P. (2009). Effect of inulin on the human gut microbiota: stimulation of *Bifidobacterium adolescentis* and *Faecalibacterium prausnitzii*. Br. J. Nutr. *101*, 541–550.

Rey, F.E., Gonzalez, M.D., Cheng, J., Wu, M., Ahern, P.P., and Gordon, J.I. (2013). Metabolic niche of a prominent sulfate-reducing human gut bacterium. Proc. Natl. Acad. Sci. U S A *110*, 13582–13587.

Robinson, M.D., McCarthy, D.J., and Smyth, G.K. (2010). edgeR: a Bioconductor package for differential expression analysis of digital gene expression data. Bioinformatics *26*, 139–140.

Ryan, K.K., Tremaroli, V., Clemmensen, C., Kovatcheva-Datchary, P., Myronovych, A., Karns, R., Wilson-Pérez, H.E., Sandoval, D.A., Kohli, R., Bäckhed, F., and Seeley, R.J. (2014). FXR is a molecular target for the effects of vertical sleeve gastrectomy. Nature *509*, 183–188.

Salonen, A., Nikkilä, J., Jalanka-Tuovinen, J., Immonen, O., Rajilić-Stojanović, M., Kekkonen, R.A., Palva, A., and de Vos, W.M. (2010). Comparative analysis of fecal DNA extraction methods with phylogenetic microarray: effective recovery of bacterial and archaeal DNA using mechanical cell lysis. J. Microbiol. Methods *81*, 127–134.

Salonen, A., Lahti, L., Salojärvi, J., Holtrop, G., Korpela, K., Duncan, S.H., Date, P., Farquharson, F., Johnstone, A.M., Lobley, G.E., et al. (2014). Impact of diet and individual variation on intestinal microbiota composition and fermentation products in obese men. ISME J. *8*, 2218–2230.

Samuel, B.S., and Gordon, J.I. (2006). A humanized gnotobiotic mouse model of host-archaeal-bacterial mutualism. Proc. Natl. Acad. Sci. U S A *103*, 10011–10016.

Scott, K.P., Martin, J.C., Campbell, G., Mayer, C.-D., and Flint, H.J. (2006). Whole-genome transcription profiling reveals genes up-regulated by growth on fucose in the human gut bacterium "*Roseburia inulinivorans*". J. Bacteriol. *188*, 4340–4349.

Scott, K.P., Martin, J.C., Chassard, C., Clerget, M., Potrykus, J., Campbell, G., Mayer, C.-D., Young, P., Rucklidge, G., Ramsay, A.G., and Flint, H.J. (2011). Substrate-driven gene expression in *Roseburia inulinivorans*: importance of inducible enzymes in the utilization of inulin and starch. Proc. Natl. Acad. Sci. U S A *108* (*Suppl 1*), 4672–4679.

Scott, K.P., Martin, J.C., Duncan, S.H., and Flint, H.J. (2014). Prebiotic stimulation of human colonic butyrate-producing bacteria and bifidobacteria, in vitro. FEMS Microbiol. Ecol. *87*, 30–40.

Sheridan, P.O., Martin, J.C., Lawley, T.D., Browne, H.P., Harris, H.M.B., Bernalier-Donadille, A., Duncan, S.H., O'Toole, P.W., Scott, K.P., and Flint, H.J. (2016). Polysaccharide utilization loci and nutritional specialization in a dominant group of butyrate-producing human colonic *Firmicutes*. Microb. Genom. *2*, e000043.

Shetty, S.A., Ritari, J., Paulin, L., Smidt, H., and De Vos, W.M. (2017). Complete genome sequence of *Eubacterium hallii* strain L2-7. Genome Announc. *5*, e01167.

Sommer, F., and Bäckhed, F. (2013). The gut microbiota—masters of host development and physiology. Nat. Rev. Microbiol. *11*, 227–238.

Sonnenburg, J.L., and Bäckhed, F. (2016). Diet-microbiota interactions as moderators of human metabolism. Nature *535*, 56–64.

Sonnenburg, J.L., Xu, J., Leip, D.D., Chen, C.-H., Westover, B.P., Weatherford, J., Buhler, J.D., and Gordon, J.I. (2005). Glycan foraging *in vivo* by an intestine-adapted bacterial symbiont. Science *307*, 1955–1959.

Sonnenburg, E.D., Smits, S.A., Tikhonov, M., Higginbottom, S.K., Wingreen, N.S., and Sonnenburg, J.L. (2016). Diet-induced extinctions in the gut microbiota compound over generations. Nature *529*, 212–215.

Sonnhammer, E.L.L., Eddy, S.R., and Durbin, R. (1997). Pfam: a comprehensive database of protein domain families based on seed alignments. Proteins *28*, 405–420.

Van Rymenant, E., Van Camp, J., Pauwels, B., Boydens, C., Vanden Daele, L., Beerens, K., Brouckaert, P., Smagghe, G., Kerimi, A., Williamson, G., et al. (2017). Ferulic acid-4-O-sulfate rather than ferulic acid relaxes arteries and lowers blood pressure in mice. J. Nutr. Biochem. *44*, 44–51.

Vangay, P., Johnson, A.J., Ward, T.L., Al-Ghalith, G.A., Shields-Cutler, R.R., Hillmann, B.M., Lucas, S.K., Beura, L.K., Thompson, E.A., Till, L.M., et al. (2018). US immigration westernizes the human gut microbiome. Cell *175*, 962–972.e10.

Vinayagam, R., Jayachandran, M., and Xu, B. (2016). Antidiabetic effects of simple phenolic acids: A comprehensive review. Phytother. Res. *30*, 184–199.

Vital, M., Howe, A., Bergeron, N., Krauss, R.M., Jansson, J.K., and Tiedje, J.M. (2018). Metagenomic insights into resistant starch degradation by human gut microbiota. Appl. Environ. Microbiol. *84*, e01562-18.

Walker, A.W., Ince, J., Duncan, S.H., Webster, L.M., Holtrop, G., Ze, X., Brown, D., Stares, M.D., Scott, P., Bergerat, A., et al. (2011). Dominant and diet-responsive groups of bacteria within the human colonic microbiota. ISME J. *5*, 220–230.

Wu, G.D., Chen, J., Hoffmann, C., Bittinger, K., Chen, Y.-Y., Keilbaugh, S.A., Bewtra, M., Knights, D., Walters, W.A., Knight, R., et al. (2011). Linking long-term dietary patterns with gut microbial enterotypes. Science *334*, 105–108.

Xu, J., Bjursell, M.K., Himrod, J., Deng, S., Carmichael, L.K., Chiang, H.C., Hooper, L.V., and Gordon, J.I. (2003). A genomic view of the human-*Bacteroides thetaiotaomicron* symbiosis. Science *299*, 2074–2076.

Ze, X., Duncan, S.H., Louis, P., and Flint, H.J. (2012). *Ruminococcus bromii* is a keystone species for the degradation of resistant starch in the human colon. ISME J. *6*, 1535–1543.

Zhang, H., Yohe, T., Huang, L., Entwistle, S., Wu, P., Yang, Z., Busk, P.K., Xu, Y., and Yin, Y. (2018). dbCAN2: a meta server for automated carbohydrate-active enzyme annotation. Nucleic Acids Res. *46* (*W1*), W95–W101.

Zhao, L., Zhang, F., Ding, X., Wu, G., Lam, Y.Y., Wang, X., Fu, H., Xue, X., Lu, C., Ma, J., et al. (2018). Gut bacteria selectively promoted by dietary fibers alleviate type 2 diabetes. Science *359*, 1151–1156.

Zmora, N., Suez, J., and Elinav, E. (2019). You are what you eat: diet, health and the gut microbiota. Nat. Rev. Gastroenterol. Hepatol. *16*, 35–56.

STAR★METHODS

KEY RESOURCES TABLE

REAGENT or RESOURCE	SOURCE	IDENTIFIER
Bacterial and Virus Strains		
Akkermansia muciniphila	Prof. Willem M. de Vos	DSM 22959
Bacteroides thetaiotaomicron	ATCC	29148
Bifidobacterium adolescentis	Dr. Karen Scott	L2-32
Collinsella aerofaciens	Dr. Karen Scott	DSM 3979
Desulfovibrio piger	Dr. Karen Scott	DSM 749
Eubacterium hallii	Dr. Karen Scott	L2-7
Eubacterium rectale	Dr. Karen Scott	A1-86
Prevotella copri	DSM	18205
Roseburia inulinivorans	Dr. Karen Scott	A2-194
Ruminococcus bromii	Dr. Karen Scott	L2-63
Chemicals, Peptides, and Recombinant Proteins		
Bacto casitone	BD	Cat#225910
Yeast extract	Oxoid	Cat#LP0021
Sodium bicarbonate	Merck	Cat#1.06329.0500
Glucose/microbiology	Merck	Cat#346351
Cellobiose	Sigma-Aldrich	Cat#22150
Starch	Sigma-Aldrich	Cat#S9765
Di-potassium hydrogen phosphate	Sigma-Aldrich	Cat#P3786
Di-hydrogen potassium phosphate	Merck	Cat#1.04873.0250
Di-ammonium sulfate	Sigma-Aldrich	Cat#A4418
Magnesium sulfate heptahydrate	Sigma-Aldrich	Cat#M7634
Calcium chloride dihydrate	Merck	Cat#1.02382.0500
Acetic acid	Sigma-Aldrich	Cat#A6283
Propionic acid	Sigma-Aldrich	Cat#P1386
n-Valeric acid	Sigma-Aldrich	Cat#240370
Isovaleric acid	Sigma-Aldrich	Cat#129542
Isobutyric acid	Sigma-Aldrich	Cat#I1754
Hemin	Sigma-Aldrich	Cat#51280
Thiamine-HCl	Sigma-Aldrich	Cat#T1270
Riboflavin	Sigma-Aldrich	Cat#R9504
Biotin	Sigma-Aldrich	Cat#B4639
Cobalamin	Sigma-Aldrich	Cat#V2876
4-Aminobenzoic acid	Sigma-Aldrich	Cat#A9878
Folic acid	Sigma-Aldrich	Cat#F8758
Pyridoxamine dihydrochloride	Sigma-Aldrich	Cat#P9158
Cysteine	Sigma-Aldrich	Cat#C7477
Resazurin	Sigma-Aldrich	Cat#R7017
Rumen fluid	Prof. Hauke Smidt	N/A
Trypticase peptone	BD	Cat#211921
Proteose peptone	Sigma-Aldrich	Cat#82450
Beef extract	BD	Cat#212303
Tween 80	Sigma-Aldrich	Cat#P4780
Vitamin K1	Sigma-Aldrich	Cat#V3501

(*Continued on next page*)

Continued		
REAGENT or RESOURCE	SOURCE	IDENTIFIER
Sodium sulfide nonahydrate	Sigma-Aldrich	Cat#208043
Sodium butyrate – $13C_4$	Sigma-Aldrich	Cat#488380
Sodium Acetate −1-13C, d3	Sigma-Aldrich	Cat#298042
Propionic acid-d6	Sigma-Aldrich	Cat#490644
Succinic acid $13C_4$ 99%	Loradan Fine Chemicals AB	Cat#CLM-1571
Sodium lactate $13C_3$ 98%	Loradan Fine Chemicals AB	Cat#CLM-1579
Succinic acid (98%)	Loradan Fine Chemicals AB	Cat#15-0400
Butyric acid	Sigma-Aldrich	Cat#19215
Sodium acetate	Sigma-Aldrich	Cat#S8750
Sodium propionate	Sigma-Aldrich	Cat#P1880
Sodium DL-lactate	Sigma-Aldrich	Cat#71720
N-tert-Butyldimethylsilyl-N-methyltrifluoroacetamide	Sigma-Aldrich	Cat#19915
Diethyl ether	Sigma-Aldrich	Cat#309958
Autoclavable Mouse Breeder Diet 5021 (Chow diet)	LabDiet	Cat#0006539
Adjusted Fat Diet (HF-HS diet)	Envigo	TD.09683
62% Sucrose diet (ZF-HS diet)	Envigo	TD.03314
EDTA	Sigma-Aldrich	Cat#EDS
TRIS	Sigma-Aldrich	Cat#76066-1KG
Ammonium acetate	Sigma-Aldrich	Cat#09688-1KG
Isopropanol	Sigma-Aldrich	Cat#I9516-500ML
Macaloid	Laguna Clay	Cat#MBENMAC
DEPC Treated water	Sigma-Aldrich	Cat#95284
RNase A Solution	QIAGEN	Cat#158922
DNase I	Roche	Cat#4716728001
UltraPure Phenol:Water (3.75:1; v/v)	Invitrogen	Cat#15594-047
UltraPure Phenol:Chloroform:Isoamyl Alcohol (25:24:1, v/v)	Invitrogen	Cat#15593-049
Chloroform:Isoamyl Alcohol (24:1, v/v)	Sigma-Aldrich	Cat#25666-100ml
Critical Commercial Assays		
1x SYBR Green Master Mix	Thermo Fisher Scientific	Cat#4309155
RNeasy Mini Kit	QIAGEN	Cat#74106
QIAmp DNA mini Kit	QIAGEN	Cat#51360
Ribo-Zero Magnetic Kit	Nordic Biolabs	Cat#MRZB12424
Agilent RNA 6000 Pico Kit	Agilent Technologies	Cat#5067-1513
ScriptSeq v2 RNA-Seq Library Preparation Kit	Nordic Biolabs	Cat#SSV21124
FailSafe Enzyme Mix	Nordic Biolabs	Cat#FSE51100
ScriptSeq Index PCR Primer	Nordic Biolabs	Cat#SSIP1234
Agencourt AMPure XP	Beckman Coulter	Cat#A63880
Deposited Data		
RNA-Seq data	This paper	SRA: PRJEB22735
PFAM	(Sonnhammer et al., 1997)	https://pfam.xfam.org/
dbCAN2	(Zhang et al., 2018)	http://cys.bios.niu.edu/dbCAN2/
Akkermansia muciniphila genome build	Ensembl bacteria	ftp://ftp.ensemblgenomes.org/pub/release-38/bacteria/fasta/bacteria_19_collection/akkermansia_muciniphila_atcc_baa_835
Bacteroides thetaiotaomicron genome build	Ensembl bacteria	ftp://ftp.ensemblgenomes.org/pub/release-38/bacteria/fasta/bacteria_0_collection/bacteroides_thetaiotaomicron_vpi_5482

(*Continued on next page*)

Continued

REAGENT or RESOURCE	SOURCE	IDENTIFIER
Bifidobacterium adolescentis genome build	Ensembl bacteria	ftp://ftp.ensemblgenomes.org/pub/release-38/bacteria/fasta/bacteria_25_collection/bifidobacterium_adolescentis_atcc_15703/
Collinsella aerofaciens genome build	Ensembl bacteria	ftp://ftp.ensemblgenomes.org/pub/release-38/bacteria/fasta/bacteria_4_collection/collinsella_aerofaciens_atcc_25986/
Desulfovibrio piger genome build	Ensembl bacteria	ftp://ftp.ensemblgenomes.org/pub/release-38/bacteria/fasta/bacteria_12_collection/desulfovibrio_piger_atcc_29098/
Eubacterium hallii genome build	Ensembl bacteria	ftp://ftp.ensemblgenomes.org/pub/release-38/bacteria/fasta/bacteria_2_collection/_eubacterium_hallii_dsm_3353/
Eubacterium rectale genome build	Ensembl bacteria	ftp://ftp.ensemblgenomes.org/pub/release-38/bacteria/fasta/bacteria_24_collection/_eubacterium_rectale_atcc_33656/
Prevotella copri genome build	Ensembl bacteria	ftp://ftp.ensemblgenomes.org/pub/release-38/bacteria/fasta/bacteria_20_collection/prevotella_copri_dsm_18205/
Roseburia inulinivorans genome build	Ensembl bacteria	ftp://ftp.ensemblgenomes.org/pub/release-38/bacteria/fasta/bacteria_20_collection/roseburia_inulinivorans_dsm_16841/
Ruminococcus bromii genome build	Ensembl bacteria	ftp://ftp.ensemblgenomes.org/pub/release-38/bacteria/fasta/bacteria_21_collection/ruminococcus_bromii_l2_63/
Experimental Models: Organisms/Strains		
Female Swiss Webster	Own breeding	N/A
Male Swiss Webster	Own breeding	N/A
Oligonucleotides		
Table S5	N/A	N/A
Software and Algorithms		
HMMER	(Eddy, 1998)	http://hmmer.org/
STAR	(Dobin et al., 2013)	https://github.com/alexdobin/STAR
Samtools	(Li et al., 2009)	http://samtools.sourceforge.net/
HTSeq	(Anders et al., 2015)	http://htseq.readthedocs.io
edgeR	(Robinson et al., 2010)	https://bioconductor.org/packages/release/bioc/html/edgeR.html
Prism (version 6)	GraphPad	N/A
Other		
CFX96 Real-Time PCR Detection System	Bio-Rad	C1000
Gas Chromatograph-Mass Spectrometer	Agilent Technologies	7890A + 5975C series
pH/Ion meter	Mettler Toledo	S220 SevenCompact
Coy chamber	COY Laboratory Products	http://coylab.com/products/anaerobic-chambers/vinyl-anaerobic-chambers/
Glucose meter	HemoCue AB	HemoCue Glucose 201+
Centrifuge	Eppendorf	5430R
Centrifuge	Thermo Scientific	Heraeus Megafuge 16R. (TX-400 Swinging Bucket Rotor)
INTELLI-MIXER with rack	LabTeamet	EL-RM-2L (EL-16mm test tube)
EchoMRI Body Composition Analyzers for Live Small Animals	Echo Medical Systems	http://www.echomri.com
PCR machine	Eppendorf	Vapo.Protect
Fast-Prep-24 Classic	MP Biomedicals	Cat#116004500

(*Continued on next page*)

Continued

REAGENT or RESOURCE	SOURCE	IDENTIFIER
Phase Lock Gel, heavy 2.0 mL tube	VWR	Cat#713-2536
Lysing matrix E		Cat#116914100
Zirconia/Silica Beads, 0.1 mm diameter	Techtum Lab AB	Cat#11079101Z
Glass beads, 3.0 mm diameter	VWR	Cat#5.1240.03
Screw cap Micro tube 2.0 mL	SARSTEDT	Cat#72.694.006
PCR plates Low 96-well black	Bio-Rad	Cat#HSP-9665
Microseal B Adhesive Sealer	Bio-Rad	Cat#MSB 1001
NanoDrop ND-1000 spectrophotometer	Thermo Scientific	http://www.nanodrop.com/Products.aspx
Bioanalyzer	Agilent Technologies	2100
Freeze dryer	LABCONCO	Freezone 4.5
Hungate-like tube	Ochs Laborbedarf	Cat#1020471

CONTACT FOR REAGENT AND RESOURCE SHARING

Further information and requests for resources and reagents should be directed to and will be fulfilled by the Lead Contact, Fredrik Bäckhed (fredrik.backhed@wlab.gu.se).

EXPERIMENTAL MODEL AND SUBJECT DETAILS

Colonization of mice with the SIM bacteria

All the mouse experiments were performed using protocols approved by the University of Gothenburg Animal Studies Committee.

For all experiments, mice were maintained in plastic gnotobiotic research isolators housed in a climate-controlled room (22°C ± 2°C) and subjected to a 12 h light/dark cycle (7:00 a.m.–7:00 p.m.) with free access to autoclaved water and food. Mice were housed in groups (n = 4-5 mice/group) according to their dietary treatment.

To colonize mice with the SIM bacteria, adult female GF Swiss Webster mice were orally gavaged twice after a 4 h fast: first, with 0.2 μl of 10^8 CFU *B. thetaiotaomicron* to obtain a more reduced gut environment, and three days later with 0.2 μl of mix of 10^8 CFU from each of the rest of the SIM strains. Mice were then bred with male GF Swiss Webster mice to generate SIM mice. Mice were bred for four generations. To characterize the colonization pattern of SIM bacteria through the gut, intestinal segments, cecal content and feces were harvested from 8-week-old male SIM mice, immediately snap-frozen in liquid nitrogen and stored at −80°C until further processed.

Colonization of mice with human feces

To generate mice colonized with human feces, fecal samples from one woman and one man were separately added to PBS buffer supplemented with reducing solution (Na_2S and cysteine dissolved in $NaHCO_3$ buffer) and transferred by oral gavage to 10-12-week-old female and male GF Swiss Webster (5 per group), respectively. The mice were colonized for 14 days and fed regular chow diet. Cecum was harvested, immediately snap-frozen in liquid nitrogen and stored at −80°C until further processed. The selected adult human donors were healthy, did not have any special dietary requirements, and had not taken any medication in the 4 months before sample donation. The participants gave informed consent and the study was approved by the Ethics Committee at Gothenburg University.

Dietary experiments with SIM mice

Adult male SIM mice aged 12-16 weeks were randomized into 3 groups (5-7 mice/group) to receive: chow (5021 rodent diet, LabDiet; fat 9% wt/wt); HF-HS (fat 20% wt/wt; sucrose 18% wt/wt, 96132 Teklad Custom Research Diet) or ZF-HS (03314 Teklad Custom Research Diet; sucrose 62% wt/wt) (Table S1). Mice were fed their respective diet *ad libitum* for 2 weeks and maintained in separate plastic gnotobiotic research isolators. At the end of the study, blood was collected from the portal vein under deep isoflurane-induced anesthesia following a 4 h fast, and intestinal segments with contents were harvested. All tissues were immediately snap-frozen in liquid nitrogen and stored at −80°C until further processed.

Bacteria strains

E. hallii L2-7 (DSM 17630), *E. rectale* A1-86 (DSM 17629), *B. adolescentis* L2-32, *C. aerofaciens* DSM 3979, *D. piger* DSM 749, *R. inulinivorans* A2-194 (DSM 16841), and *R. bromii* L2-63 were obtained from Dr Karen Scott, the Rowett Institute of Nutrition and Health, Aberdeen, UK. *B. thetaiotaomicron* ATCC 29148 and *P. copri* DSM 18205 were obtained from ATCC and DSMZ,

respectively. *A. muciniphila* DSM 22959 was obtained from Professor Willem de Vos, Laboratory of Microbiology, Wageningen University, the Netherlands. All strains in this study originate from human feces.

E. hallii, E. rectale, B. adolescentis, B. thetaiotaomicron, C. earofaciens, R. inulinivorans were maintained in Hungate-like tube (Ochs Laborbedarf, Germany) under CO_2 in YCFA medium (Ze et al., 2012). *R. bromii* was maintained in Hungate-like tubes in M2GSC media supplemented with rumen fluid (Miyazaki et al., 1997). *D. piger* was maintained in Hungate-like tubes in CO_2 in YCFA medium supplemented with 30 mM lactate. *P. copri* was grown in PYG medium (Media 104 in the DSMZ catalog), and *A. muciniphila* was grown in a basal mucin-based media (Derrien et al., 2004). All bacteria were cultured anaerobically in 15 mL Hungate tubes at 37°C in a Coy chamber.

METHOD DETAILS

Body composition and glucose tolerance test

Body composition (including liver fat) was determined using magnetic resonance imaging (EchoMRI, Houston, TX). For the glucose tolerance test, mice were fasted for 4 h before intraperitoneal injection of 30% glucose solution (1.5 mg/g body weight). Blood glucose levels from tail vein were measured at baseline, and after 15, 30, 60, 90 and 120 min.

Genomic DNA extraction and 16S rRNA quantitative PCR

After isolation of genomic DNA from intestinal segments, cecal content and feces using repeated bead beating (Salonen et al., 2010), 16S rRNA quantitative PCR was performed with a CFX96 Real-Time System (Bio-Rad Laboratories). Samples were analyzed in a 25 μL reaction mix consisting of 12.5 μL 1xSYBR Green Master Mix buffer (Thermo Scientific, Waltham, Massachusetts, USA), 0.2 μM of each primer and 5 μL of template DNA, water or genomic DNA extracted from feces or cecal content. Standard curves of 16S rRNA PCR product of *E. hallii*, *E. rectale*, *B. adolescentis*, *C. aerofaciens*, *D. piger*, *R. inulinivorans*, *R. bromii, B. thetaiotaomicron*, *P. copri* and *A. muciniphila* were created using serial 10-fold dilution of the purified PCR product. qPCR was performed as reported previously: for *E. hallii*, *E. rectale*, *B. adolescentis*, *R. inulinivorans*, *R. bromii* and total bacteria (Ramirez-Farias et al., 2009); for *C. aerofaciens* (Kageyama et al., 2000); for *D. piger* (Fite et al., 2004); for *P. copri* (Matsuki et al., 2002); for *B. thetaiotaomicron* (Samuel and Gordon, 2006); and for *A. muciniphila* (Collado et al., 2007) (Table S5). All reactions were performed in duplicate in one run and in duplicate PCR runs. The data are expressed as log of 16S rRNA copy per g of luminal content or feces.

Quantification of SCFAs and organic acids

SCFAs and organic acids in the cecum were measured by gas chromatography. For the extractions, 100-250 mg of frozen cecal content was transferred to a glass tube (16x125 mm) fitted with a screw cap and 100 μL of stock solution of internal standard (1 M [1-^{13}C] acetate, 0.2 M [$^2H_6$]propionate and [$^{13}C_4$]butyrate, 0.5 M [^{13}C]lactate and 40 mM [$^{13}C_4$]succinic acid) was added. Prior to extraction, samples were freeze-dried at −50 °C for 3 h (yield 40–98 mg dry weight). The extraction and quantification of SCFAs and organic acids was performed as previously reported (Ryan et al., 2014).

Microbial RNA extraction

Approximately 100 mg frozen cecum content was re-suspended in 500 μL ice-cold TE buffer (Tris–HCl pH 7.6, EDTA pH 8.0). Total RNA was extracted from the re-suspended cell pellet according to the Macaloid-based RNA isolation protocol (Egert et al., 2007) with the use of Phase Lock Gel heavy (5 Prime, Hamburg) (Murphy and Hellwig, 1996) during phase separation. The aqueous phase was purified using the RNAeasy mini kit (QIAGEN, USA), including an on-column DNaseI (Roche, Germany) treatment as described previously (Egert et al., 2007). Total RNA was eluted in 30 μL ice-cold TE buffer and the RNA quantity and quality were assessed using a NanoDrop ND-1000 spectrophotometer (Nanodrop Technologies, Wilmington, USA).

Metatranscriptome analysis of the SIM community

The metatranscriptome datasets were generated by Illumina sequencing of 15 cDNA libraries derived from mRNA enriched samples of the cecal SIM microbiota. The mRNA enrichment was performed by the removal of 16S and 23S rRNA using sequence-based capture probes attached to magnetic beads [Ribo-ZeroTM Magnetic Kit (Bacteria), Epicenter, Illumina] using the manufacturer's protocols. The enriched mRNA was quantified spectrophotometrically (NanoDrop) and its quality was assessed using an Agilent 2100 Bioanalyzer (Agilent Technologies). Thirty Illumina sequencing libraries were constructed from double-stranded cDNA prepared after RNA fragmentation according to the manufacturer's protocols (ScriptSeq v2 RNA-Seq Library preparation Kit, Epicenter, Illumina). Each sequencing library was barcoded (ScriptSeqTM Index PCR Primers, Epicenter, Illumina). Sequencing was performed at the Genomics Core Facility of the Gothenburg University using Illumina Hiseq2000, which generates on average ∼15 million reads for each sample. The reads were trimmed from adaptor sequences and quality (Phred quality score > 30), then simultaneously aligned to the corresponding bacterial genome of SIM using STAR 2.3.1u (Dobin et al., 2013). The resulting bam-files were indexed and sorted using Samtools 0.1.18 (Li et al., 2009). Gene counts were calculated using HTSeq-count 0.5.4p3 (Anders et al., 2015) based on the transcript annotation and globally normalized using a standard negative binomial approach through the R-package edgeR (Robinson et al., 2010). Circos was used to plot the genome coverage and the comparative analyses of differentially expressed genes (Krzywinski et al., 2009).

The genes were functionally annotated using KEGG orthology (KO), CAZymes and Pfam. We annotated CAZymes based on the HMMER, DIAMOND and Hotpep tools of dbCAN2 meta-server (Zhang et al., 2018) using the following: E-value < 1e-15, coverage > 0.35 for HMMER; E-value < 1e-102, hits per query (-k) = 1 for DIAMOND; and frequency > 6.0, hits > 2.6 for Hotpep. We annotated Pfam domains using HMM profiles of Pfam Database (version 31) by HMMER (default E-value, 10) (Eddy, 1998).

Metabolomic analysis of plasma from SIM mice

Aliquots of portal vein plasma from SIM mice fed chow, HF-HS or ZF-HS diets were analyzed by untargeted liquid chromatography - mass spectrometry (LC-MS) and gas chromatography - mass spectrometry (GC-MS) at Metabolon (Durham, USA).

QUANTIFICATION AND STATISTICAL ANALYSIS

Values are presented as means ± SEM. For graph plotting and statistical analysis we used GraphPad Prism (version 6, GraphPad Software, San Diego, CA, USA) unless otherwise indicated. Statistical comparison of two groups was performed by Student's t test, comparisons of three or more groups were performed by one-way analysis of variance (ANOVA) and corrected for multiple comparison with Tukey post-tests.

DATA AND SOFTWARE AVAILABILITY

The RNA-seq data reported in this paper have been deposited in the ENA sequence read archive (http://www.ebi.ac.uk/ena/data/view) under accession number PRJEB22735.

Cell Reports, Volume *26*

Supplemental Information

Simplified Intestinal Microbiota to Study

Microbe-Diet-Host Interactions in a Mouse Model

Petia Kovatcheva-Datchary, Saeed Shoaie, Sunjae Lee, Annika Wahlström, Intawat Nookaew, Anna Hallen, Rosie Perkins, Jens Nielsen, and Fredrik Bäckhed

Table S1. Nutritional information of the three mouse diets. Related to STAR methods.

Ingredients (% by weight)	Chow* (Lab Diet 5021)	HF/HS** (Harlan TD.96132)	ZF/HS** (Harlan TD.03314)
**Protein**	**21.0**	**20.9**	**20.0**
**Fat**	**9.4**	**20.2**	**0**
Saturated fatty acids	2.7	8.3	-
Trans fatty acids	-	3.4	-
Monounsaturated fatty acids	2.6	7.1	-
Polyunsaturated fatty acids	4.1	1.4	-
**Carbohydrates*** **	**33.2**	**46.3**	**62.8**
Starch	32.1	16.0	-
Sucrose	0.7	18.3	62.8
Maltodextrin	-	12.0	-
Glucose	0.2	-	-
Fructose	0.2	-	-
**Neutral detergent fiber**	**14.8**	**4.0**	**11.0**
Hemicellulose	10.2	-	-
Cellulose	4.6 ****	4.0	11.0

*Natural ingredient diet; **Purified diet; ***Available carbohydrates; ****Cellulose and lignin.

Table S5. Oligonucleotides, related to KEY RESOURCES TABLE

REAGENT or RESOURCE	SOURCE	IDENTIFIER
Oligonucleotides		
AM1	Sigma-Aldrich	CAGCACGTGAAGGTGGGGAC
AM2	Sigma-Aldrich	CCTTGCGGTTGGCTTCAGAT
Bt1F	Sigma-Aldrich	ATAGCCTTTCGAAAGRAAGAT
Bt1R	Sigma-Aldrich	CCAGTATCAACTGCAATTTTA
Bif164F	Sigma-Aldrich	GGGTGGTAATGCCGGATG
BiADO-2	Sigma-Aldrich	CGAAGGGCTTGCTCCCAGT
AERO-F	Sigma-Aldrich	CTTTCAGCAGGGAAGAGTCAA
AERO-R	Sigma-Aldrich	AGCCATGCACCACCTGTATGG
DSV691-F	Sigma-Aldrich	CCGTAGATATCTGGAGGAACATCAG
DSV826-R	Sigma-Aldrich	ACATCTAGCATCCATCGTTTACAGC
EhalF	Sigma-Aldrich	GCGTAGGTGGCAGTGCAA
EhalR	Sigma-Aldrich	GCACCGRAGCCTATACGG
RrecF	Sigma-Aldrich	GCGGTRCGGCAAGTCTGA
Rrec630mR	Sigma-Aldrich	CCTCCGACACTCTAGTMCGAC
g-Prevo-F	Sigma-Aldrich	CACRGTAAACGATGGATGCC
g-Prevo-R	Sigma-Aldrich	GGTCGGGTTGCAGACC
RrecRi630F	Sigma-Aldrich	GTCATCTAGAGTGTCGGAGG
Erec870R	Sigma-Aldrich	AGTTTYATTCTTGCGAACG
Rflbr730F	Sigma-Aldrich	GGCGGCYTRCTGGGCTTT
Clep866mR	Sigma-Aldrich	CCAGGTGGATWACTTATTGTGTTAA
UniF	Sigma-Aldrich	GTGSTGCAYGGYYGTCGTCA
UniR	Sigma-Aldrich	ACGTCRTCCMCNCCTTCCTC
27F	Sigma-Aldrich	GTTTGATCCTGGCTCAG
1492 R	Sigma-Aldrich	CGGCTACCTTGTTACGAC
